# Differential activation of NF-Κβ and HIF-1α between fetal liver-derived and myeloid-derived macrophages drives inflammatory differences following *Mycobacterium abscessus* infection

**DOI:** 10.1128/iai.00136-26

**Published:** 2026-06-15

**Authors:** Haleigh N. Gilliland, Soledad Soverina, Kayla N. Conner, Taryn E. Vielma, Andrew J. Olive

**Affiliations:** 1 Department of Microbiology, Genetics, and Immunology, College of Osteopathic Medicine, Michigan State University, East Lansing, Michigan, USA; Rutgers New Jersey Medical School 12286, Newark, New Jersey, USA

**Keywords:** *Mycobacterium*, nontuberculous *Mycobacterium*, alveolar macrophages, innate immunity

## Abstract

Pulmonary infections caused by *Mycobacterium abscessus* (Mab)*,* a rapidly growing nontuberculous mycobacterium (NTM), are rising in patients with preexisting lung disease. Unfortunately, the natural recalcitrance of Mab to antibiotics drives high rates of treatment failures. Understanding the initial host-pathogen interactions that result in Mab control or disease is critical to identify new therapeutic targets. Lung macrophages are the first immune cells that Mab encounters, yet how distinct macrophage subsets, including resident fetal liver-derived alveolar macrophages (AMs) and recruited myeloid-derived macrophages, differentially respond to and control Mab remains unknown. Using primary AMs or fetal-liver derived alveolar macrophages (FLAMs) as a model of AMs and bone marrow macrophages (BMDMs) as a model of recruited myeloid macrophages, we found distinct macrophage subsets that similarly control intracellular Mab in resting and interferon-gamma (IFNγ)-activated cells with similar levels of cell death. However, divergent inflammatory responses were observed with resting BMDMs robustly activating NF-κB-dependent proinflammatory cytokines, while FLAMs only transiently induced this key inflammatory signature. We further found that Mab-infected IFNγ activated FLAMs and did not robustly induce HIF1α, resulting in reduced Nos2 expression. Thus, differences in the activation of transcription factors in fetal liver- and myeloid-derived macrophages drives distinct host responses against the same Mab pathogen. Our results provide a new understanding of early interactions with Mab in the lungs and suggest differences that distinct macrophage subsets may contribute to susceptibility to Mab pulmonary disease.

## INTRODUCTION

Pulmonary infections caused by rapidly growing nontuberculous mycobacterium (NTM) are on the rise, with a 400% increase in prevalence from 1987 to 2015 (1–3). Of these infections, those caused by *Mycobacterium abscessus* (Mab) are the second most common (4). While the majority of immunocompetent hosts control Mab infections, patients with pre-existing pulmonary conditions, including cystic fibrosis (CF), chronic obstructive pulmonary disease (COPD), or bronchiectasis, are at a particularly high risk for chronic Mab pulmonary infection (5–8). Complicating matters, Mab remains intrinsically resistant to many antibiotics, requiring multidrug treatment regimens and high rates of treatment failure (9–11). The lungs of susceptible patient cohorts are characterized by structural damage, inflammation, and/or changes in mucus regulation, changing the pulmonary environment in dramatic ways (7, 12, 13). How these structural and inflammatory changes directly alter Mab-host interactions remains unclear. With no protective vaccine and limited drug options, understanding how immunocompetent hosts control Mab is of critical importance to develop more clinically relevant host-directed therapies.

Central to host-pathogen interactions in the lungs are macrophages, key innate immune cells tasked with sensing the environment, initiating inflammation, and controlling infections (14, 15). In the lungs, several macrophage sub-populations, including fetal-derived resident alveolar macrophages (AMs) and myeloid-derived interstitial/recruited macrophages, play important roles in maintaining pulmonary homeostasis and protecting against respiratory pathogens (15–17). The difference in ontogeny and the local environment drives important phenotypic differences in these distinct macrophage subtypes (14, 16). Resident lung AMs are maintained in the airspace to recycle surfactants produced by lung epithelial cells (15–17). AMs are the first immune cells to detect inhaled pathogens and control initial immune responses during pulmonary infection, including Mab (18). Works examining other respiratory infections, including *Mycobacterium tuberculosis*, suggest that AMs are hypo-inflammatory and restrain their interactions with T cells to prevent robust adaptive immune activation (19, 20). In contrast, myeloid-derived macrophages are highly inflammatory cells that drive increased pathogen control and T cell activation (19, 21, 22). Combined, these inflammatory responses result in tissue inflammation that modulates macrophage function.

During lung infections, inflammatory signals can further modify the local environment to drive protection or pathology (23). One key inflammatory cue is the production of the cytokine interferon-gamma (IFNγ) (24). This protective cytokine is primarily produced by NK and Th1-activated T cells and is required for humans to control mycobacterial infections as the loss of IFNγ signaling results in Mendelian susceptibility to mycobacterial disease (MSMD) (25, 26). IFNγ contributes to immune control by upregulating antimicrobial and T cell-mediated restriction pathways (20, 27). These pathways include the production of reactive oxygen species (ROS) and nitric oxide (NO) that directly control pathogens and inflammatory signaling (26, 27). Recent work suggests IFNγ responses are distinct in different macrophage subtypes, with these differences driving alternative immune functions (19, 28). Given that patients who are susceptible to Mab are characterized by chronic inflammatory lung environments, it is important to understand how distinct macrophage subsets respond to Mab in both resting and inflammatory states.

To date, the majority of studies examining Mab-macrophage interactions have used murine bone marrow-derived macrophages (BMDMs) and/or immortalized macrophage cell lines including RAW264.7, J774, and Thp1 cells (29–32). These studies identified important host genes required for the uptake of Mab as well as roles for TLR2 and Nod2 in activating pathways that drive TNF, type I IFN, and NO production to control Mab (29, 33). Mab-infected zebrafish embryo studies have found TNF signaling and IL8-mediated neutrophil recruitment are required for granuloma formation (34, 35). However, we continue to lack an understanding of the early interactions between Mab and AMs. One reason for this gap in knowledge is the limited approaches available to understand AMs. AMs are particularly challenging to isolate and to maintain in the AM-like states observed in the lungs. Recent advances in *ex vivo* culturing now enable a mechanistic understanding of interactions with AM-like cells (36–38). One model we recently developed is fetal liver-derived alveolar-like macrophages (FLAMs) (36). These cells are isolated from the fetal liver, which is the source of AMs *in vivo*, and cultured with two key lung cytokines, GM-CSF and TGFβ (36, 39, 40). FLAMs are transcriptionally similar to AMs yet distinct from myeloid-derived BMDMs in both resting and IFNγ-activated states (19), thus serving as a useful model to understand mechanistic AM responses during infection. Recent work also suggests that FLAMs recapitulate many transcriptional characteristics observed in AMs *in vivo* (41).

To fill this gap in knowledge here, we defined macrophage subset-specific early host responses to Mab infection. We first compared the uptake and bacterial growth kinetics in BMDMs and FLAMs to model distinct populations in the lungs in both resting and IFNγ-activated cells, finding few differences in Mab control or cell death early following infection. In contrast, we observed significant differences in the innate response in resting and IFNγ-activated FLAMs and BMDMs that were dependent on Mab infection. Our mechanistic studies found differences in the activity of key transcription factors including NF-κB and HIF1α that we found partially mediate the differential inflammatory response between FLAMs and BMDMs. These data uncover key differences in the transcriptional response of distinct macrophage subtypes, identifying cell type-specific inflammation that may play an important role in regulating disease control or progression during respiratory infections.

## RESULTS

### Mab persists in BMDMs and FLAMs over 48 h of infection

**Fig 1:**
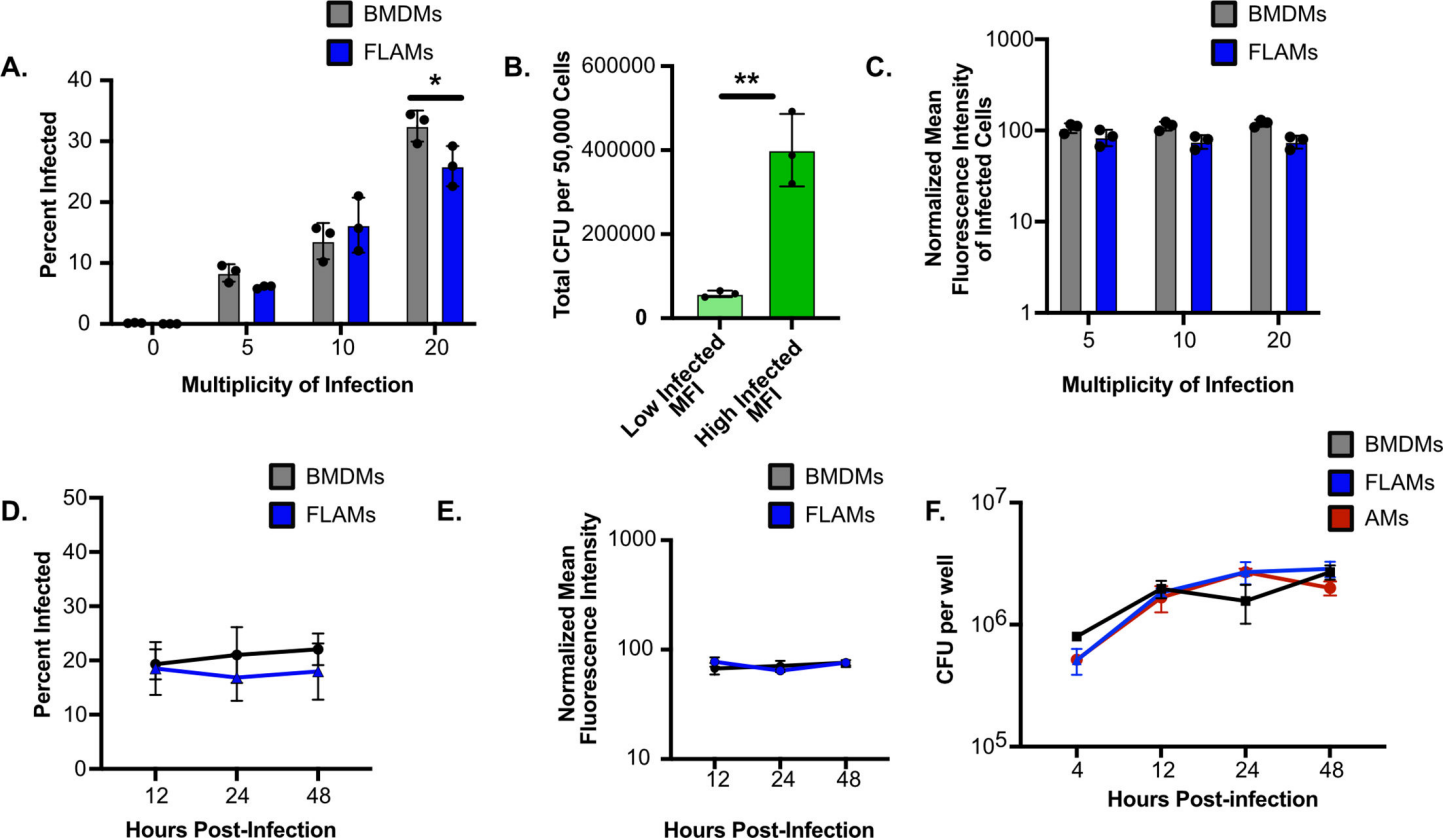
AMs, FLAMs, and BMDMs similarly take up and control intracellular Mab. (**A**) BMDMs or FLAMs were infected for 4 h with the indicated MOI of mEmerald-Mab, and flow cytometry was used to quantify the percent of cells infected. (**B**) Immortalized BMDMs (iBMDMs) were infected with mEmerald-Mab for 3 days, and 50,000 infected cells with either high mEmerald mean fluorescence intensity (MFI) or low MFI were sorted, plated, and total CFU from each population was quantified. (**C**) BMDMs or FLAMs were infected for 4 h with the indicated MOI of mEmerald-Mab, and flow cytometry was used to quantify the MFI of infected cells. (**D**) BMDMs or FLAMs were infected for 4 h with mEmerald-Mab (MOI 5), and flow cytometry was conducted at the indicated time points post-infection to quantify the percent of cells infected and (**E**) the MFI of infected cells from each condition. (**F**) BMDMs, FLAMs, or primary AMs were infected for 4 h with mEmerald-Mab (MOI 5), then cells were lysed, and intracellular Mab was quantified by CFU plating at the indicated time points. Each experiment is representative of at least three independent experiments with at least three biological replicates per experiment. *P < 0.05; **P < 0.01 by two-way analysis of variance (ANOVA) with Tukey’s correction for multiple comparisons (**A and F**) and unpaired t-test for B. All significant comparisons are indicated, and remaining comparisons are not significant.

As a first step to understand differences in the early host-Mab interactions between macrophage subsets, we compared whether BMDMs or FLAMs phagocytose Mab with different efficiency. To test this, we infected resting BMDMs derived from HoxB8-mediated conditionally immortalized progenitors or FLAMs with the smooth Mab strain ATCC19977 expressing constitutively active mEmerald GFP at increasing multiplicities of infection (MOIs) (30). Four hours later, flow cytometry was used to quantify the percentage of cells that were GFP-positive. While we noted an increase in the percentage of infected cells as the MOI increased, we did not observe any significant differences in the uptake between BMDMs and FLAMs (Fig. 1A). In addition to the percentage of infected cells, we wondered whether the mean fluorescence intensity (MFI) of infected cells could be used as a surrogate for the intracellular bacterial level. To test this prediction, we first infected immortalized BMDMs with mEmerald-Mab and used cell sorting to isolate infected cells with high or low GFP MFI and plated for colony-forming unit (CFU) (Fig. 1B). We found that cells sorted from the high-GFP MFI contained more Mab on a per-cell basis than the same number of cells from the low-GFP group (Fig. 1C). These data show that MFI is a useful correlate of intracellular bacterial levels. When we examined the MFI of cells infected with increasing MOI of Mab at 4 h, we observed no significant differences between BMDMs and FLAMs (Fig. 1D). These data show that the uptake of Mab by BMDMs and FLAMs is similar.

We next characterized the intracellular dynamics of Mab in distinct macrophage subsets over time. BMDMs or FLAMs were infected with mEmerald-Mab at an MOI of 5, and 12, 24, and 48 h later, flow cytometry was used to monitor intracellular Mab. We observed no significant changes in the percent of infected cells or the MFI of infected cells over this time (Fig. 1D and E). To confirm these results, this experiment was repeated, and primary AMs from bronchial lavage fluid were included. Cells were infected and then lysed over time up to 48 h following infection, followed by quantification of colony-forming units (CFU) (Fig. 1F). We found no significant differences in intracellular Mab between cell types over the entire time course. These data suggest that Mab persists in resting BMDMs and FLAMs over several days, with similar bacterial uptake and intracellular burdens in the first 48 h following infection.

### Mab infection of FLAMs and AMs results in distinct cytokine production independent of cell death

**Fig 2:**
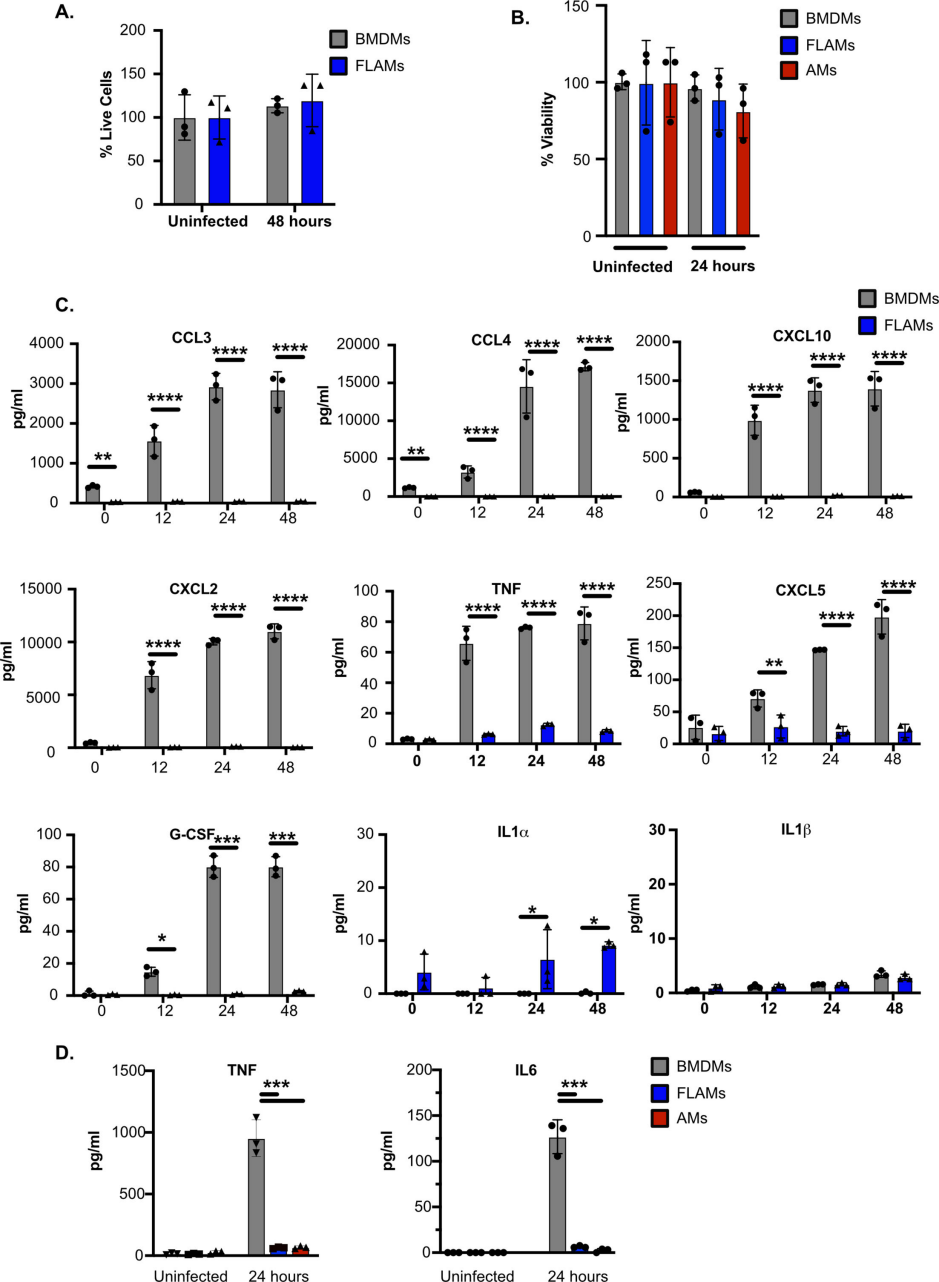
BMDMs are more inflammatory than AMs and FLAMs in response to Mab infection without driving more cell death. (**A**) Shown is the percent of viable cells (Live/Dead stain negative) quantified by flow cytometry for BMDMs or FLAMs infected with mEmerald-Mab (MOI 5) for 48 h. (**B**) The percent of viable cells 24 h following mEmerald-Mab infection of BMDMs, FLAMs, or primary AMs (MOI 5) was quantified using CellTiter-Glo. The percent of cells that were alive was determined by normalizing each sample to the mean of the uninfected cell type control. (**C**) Shown is the concentration of cytokines from the supernatants of BMDMs or FLAMs infected with mEmerald-Mab (MOI 5) at the indicated time points. Panels **A and B** are representative of three independent experiments with three biological replicates per group. Panel** C** is from a single multiplex experiment with three biological replicates per group. (**D**) BMDMs, FLAMs, or primary AMs were infected with mEmerald-Mab for 24 h. TNF and IL-6 were then quantified from the supernatants by ELISA. *P < 0.05, **P < 0.01, ***P < 0.001, and ****P < 0.0001 by two-way ANOVA with Tukey’s correction for multiple comparisons.

Given the similar intracellular Mab dynamics between BMDMs, AMs, and FLAMs, we next tested whether there are differences in cell death and inflammation during infection. FLAMs and BMDMs were infected with Mab at an MOI of 5, and 48 h later, cell death was quantified by flow cytometry using a viability dye (Fig. 2A) (42). Our results found limited induction of cell death following infection in either cell type using flow cytometry. To confirm these results, we next quantified total ATP via a CellTiter-Glo assay as an orthologous cell death approach and tested primary AMs, FLAMs, and primary BMDMs (Fig. 2B). Our results showed no significant differences in death between any cell type 24 h following infection. These data suggest that, over the first day of infection, when the intracellular levels of Mab are similar between BMDMs, AMs, and FLAMs, there are only minor changes in cell viability.

We next examined whether the inflammatory response was different between Mab-infected BMDMs and FLAMs. Cells were infected with Mab at an MOI of 5, and 12, 24, and 48 h later, proinflammatory cytokines were quantified in the cell culture supernatants by a multiplex Luminex assay. We observed significant differences between BMDMs and FLAMs, with BMDMs producing 100–10,000 times the levels of the cytokines CCL3, CCL4, CXCL2, CXCL5, CXCL10, TNF, and G-CSF compared to FLAMs (Fig. 2C). We noted a small but significant 5-fold induction of IL-1α only in FLAMs infected for 24 and 48 h. However, we observed no IL-1β was produced by either macrophage subset. To validate these results, we infected primary AMs in addition to FLAMs and BMDMs and then quantified TNF and IL-6 secretion the following day. Both AMs and FLAMs secreted significantly lower levels of TNF and IL-6 compared to BMDMs (Fig. 2D). Taken together, these results show that FLAMs are a robust model of the inflammatory response of primary AMs and Mab-infected BMDMs have significantly higher and broader inflammatory responses independently of bacterial levels or cell death.

### Transcriptional analysis finds that Mab infection of BMDMs is more inflammatory than FLAMs

**Fig 3:**
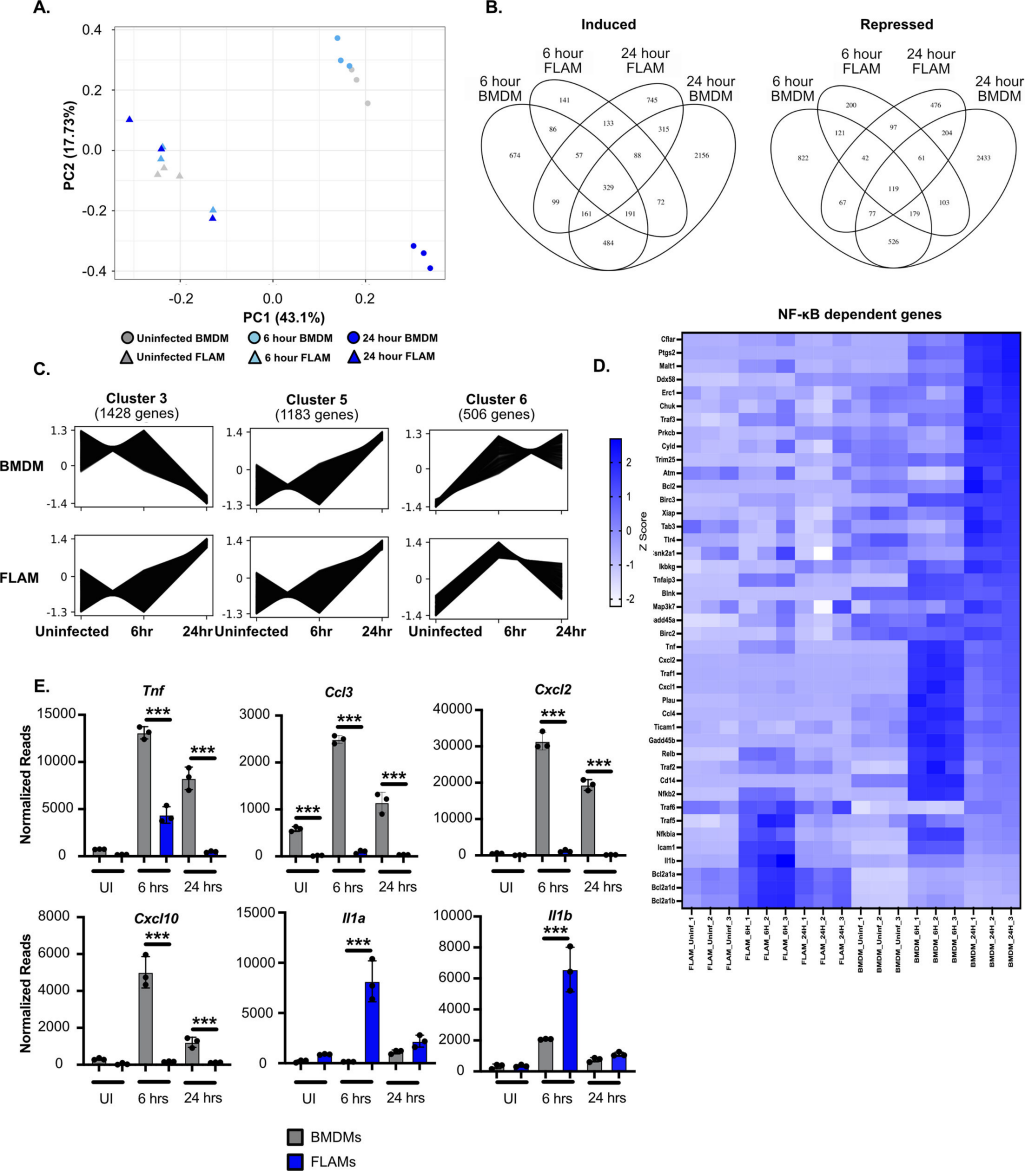
BMDMs induce a stronger NF-κB transcriptional signature during Mab infection compared to FLAMs. (**A**) A principal component analysis (PCA) plot comparing the transcriptional responses of BMDMs and FLAMs during Mab infection. (**B**) Venn diagrams showing shared and unique genes that were significantly induced or repressed in BMDMs and FLAMs during Mab infection and identified using DESeq2. (**C**) Representative clusters of genes that respond similarly to Mab infection in BMDMs and FLAMs over time. (**D**) Normalized counts of NF-κB genes from the KEGG pathway set were compared across samples of BMDMs and FLAMs infected with Mab over time and are expressed as a heatmap. The color scale represents the z-score calculated from normalized read counts across samples for each gene. (**E**) Normalized counts of a subset of NF-κB genes that were differentially regulated in BMDMs (gray) and FLAMs (blue) during Mab infection. ***P < 0.001 based on adjusted *P*-values using DESeq2 comparisons.

To better understand how Mab infection alters the transcriptional landscape, we next characterized changes to the global transcriptomes of both FLAMs and primary BMDMs during Mab infection. Cells were infected with Mab at an MOI of 5, and then 6 and 24 h later, when the intracellular Mab burden is identical, RNA was isolated, and bulk RNA sequencing analysis was performed (Table S1). Principal component analysis (PCA) showed a strong separation between BMDMs and FLAMs in all conditions along PC1, in line with our previous studies (Fig. 3A) (19). While there was a major shift in BMDMs at 24 h following infection along PC2, this was not observed in FLAMs. This suggests that Mab infection induces less dramatic changes to the transcriptome of FLAMs compared to BMDMs. We next compared genes that were differentially expressed in either FLAMs or BMDMs over time (Fig. 3B). We noted 329 and 119 genes were induced or repressed, respectively, in both FLAMs and BMDMs at all time points. We observed that there were four times more uniquely induced or repressed genes in BMDMs at 6 h post-infection compared to FLAMs, a trend that remained at 24 h post-infection. To identify key differences in transcriptional activation during Mab infection of BMDMs and FLAMs, we used clustering analysis to group genes whose expression changes similarly across all conditions. We identified seven unique clusters and conducted pathway and transcription factor analysis to identify transcriptional networks that were associated with each cluster (Fig. 3C and Table S2). Of note, we found that Cluster 3 contained genes that were uniquely induced during Mab infection in FLAMs. Kyoto Encyclopedia of Genes and Genomes (KEGG) pathway analysis found significant enrichment of genes associated with metabolism, including glycine, serine, and threonine metabolism, as well as peroxisomes, a key hub of lipid metabolism. Cluster 5 contained genes that were induced in both FLAMs and BMDMs and was enriched for pathways related to the lysosome and phagosome, as well as antigen presentation. In contrast, Cluster 6 contained genes that were uniquely induced in BMDMs and was enriched for inflammatory pathways, including NF-κΒ and TNF. Transcription factor analysis of Cluster 6 agreed with the KEGG pathway analysis and identified a strong NF-κΒ signature. When we more closely examined a subset of NF-κΒ-related genes, we found that in BMDMs, many of these genes were more highly induced, and the expression levels of these pro-inflammatory transcripts were higher (Fig. 3D). When we examined a subset of chemokines that were elevated in our multiplex analysis above, we noted a high, persistent expression of each in BMDMs relative to FLAMs (Fig. 3E). The exception to this pattern was IL-1α and IL-1β. These cytokines were robustly induced at high levels in FLAMs compared to BMDMs, suggesting unique regulation of IL1 in FLAMs. Taken together, our transcriptional analysis found that while both FLAMs and BMDMs sense and respond to Mab infection, BMDMs are more inflammatory than FLAMs.

### Differential Nrf2 and NF-κB activation partially contributes to differences in inflammation of FLAMs and BMDMs during Mab infection

**Fig 4:**
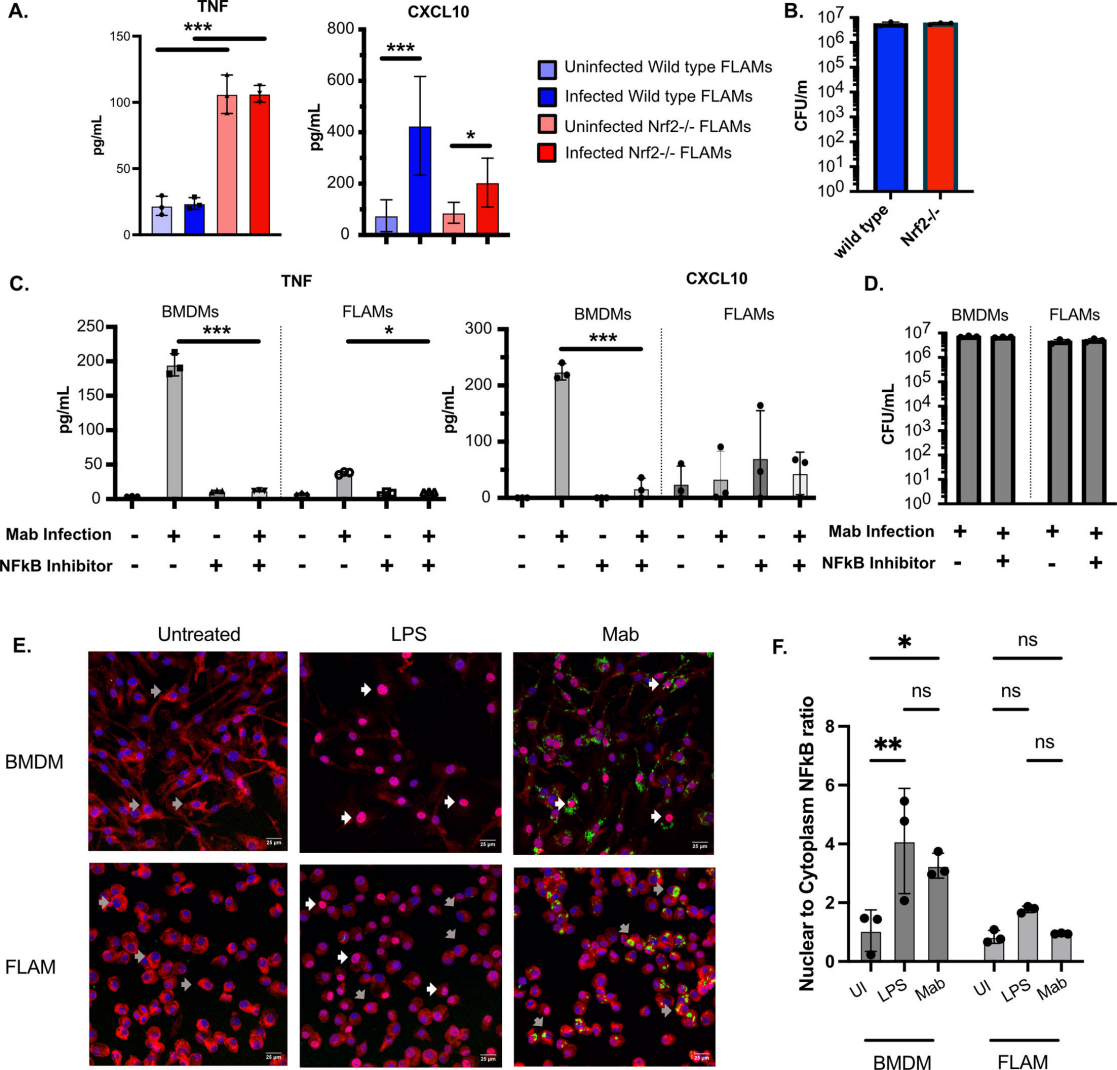
Inhibition of NF-κB equalizes the inflammatory response of BMDMs and FLAMs to Mab infection. (**A**) Shown is the concentration of TNF and CXCL10 from the supernatants of wild-type or Nrf2^−/−^ FLAMs infected with mEmerald-Mab (MOI 5) for 48 h by enzyme-linked immunosorbent assay (ELISA). (**B**) CFU enumeration from wild-type or Nrf2^−/−^ FLAMs at 48 h. (**C**) Shown is the concentration of TNF and CXCL10 from the supernatants of BMDMs or FLAMs infected with mEmerald-Mab (MOI 5) for 48 h in the presence and absence of the NF-κB inhibitor (5 μM). (**D**) CFU enumeration of Mab from BMDMs or FLAMs at 48 h in the presence and absence of the NF-κB inhibitor (5 μM). (**E**) The indicated cells were left untreated/uninfected (UI), with lipopolysaccharide (LPS) (10 ng/mL), or infected with Mab-mEmerald for 6 h. Shown are representative micrographs showing the localization of the nucleus (blue), NF-κB p65 (red), and Mab-mEmerald (green). White arrowheads indicate examples of translocated NF-κB in the nucleus. Gray arrowheads indicate cytosolic NF-κB. Scale bars indicate 25 uM in each micrograph. (**F**) Shown is the quantification of data by analyzing micrographs from C and using CellProfiler to quantify the mean fluorescence intensity (MFI) of NF-κB, that is, nuclear versus cytosolic. For conditions with Mab quantification only includes infected cells and excludes bystanders. All data are representative of two or three independent experiments with at least three biological replicates. ns: not significant, *P < 0.05, **P < 0.01, ***P < 0.001 by two-way ANOVA with Tukey’s correction for multiple comparisons. All significant comparisons are indicated, and remaining comparisons are not significant.

We next examined possible mechanisms contributing to the reduced inflammation we observed in FLAMs following Mab infection. Previous work with *Mycobacterium tuberculosis* infection found that activation of the transcription factor Nrf2 inhibited inflammatory pathways in AMs (43). To directly test if Nrf2 was responsible for FLAMs’ hypo-inflammatory response, we infected wild-type and Nrf2^−/−^ FLAMs with Mab and examined proinflammatory cytokine production (Fig. 4A). We found that in uninfected resting Nrf2^−/−^ FLAMs, there was an increase in the baseline production of TNF compared to wild-type cells. Interestingly, we observed no Mab-dependent differences in these cytokines following infection nor any difference in bacterial levels (Fig. 4B). These data suggest that while Nrf2 modulates the baseline expression of some inflammatory cytokines in FLAMs, it does not suppress inflammatory signaling in response to Mab infection.

Our transcriptional analysis suggested that BMDMs induce NF-κB pathways more robustly than FLAMs. To examine the role of NF-κB in the hyper-inflammatory response of BMDMs, we inhibited NF-κB using the chemical inhibitor BAY 11-7082 in both FLAMs and BMDMs and quantified TNF, an NF-κB-dependent transcript, by ELISA 24 h following Mab infection (Fig. 4C) (44). We observed that blocking NF-κB in Mab-infected BMDMs led to a 20-fold decrease in TNF, resulting in a cytokine level that was similar to infected NF-κB-blocked FLAMs. Critically, Mab levels were unchanged (Fig. 4D). These data suggest that NF-κB activation in BMDMs drives higher levels of inflammatory cytokines than FLAMs during Mab infection.

To directly examine the activation of NF-κB in FLAMs and BMDMs, we used immunofluorescence microscopy to quantify the nuclear translocation of the NF-κB p65 subunit. FLAMs and BMDMs were left untreated, stimulated with LPS, or infected with Mab at an MOI of 5 for 6 h. Using CellProfiler, we quantified the ratio of NF-κB in the nucleus to the cytosol based on the MFI of staining in each cell (Fig. 4E). In BMDMs, both LPS and Mab infection resulted in a dramatic translocation of NF-κB to the nucleus, suggesting robust activation of this transcription factor. In contrast, we observed no significant changes in NF-κB p65 localization in FLAMs following either LPS stimulation or Mab infection (Fig. 4F). These data show that in FLAMs, NF-κB is not robustly activated following LPS stimulation or Mab infection, which contributes to the hypoinflammatory response observed in our transcriptomics and cytokine profiling.

### Activating BMDMs or FLAMs with IFNγ does not alter Mab control

**Fig 5:**
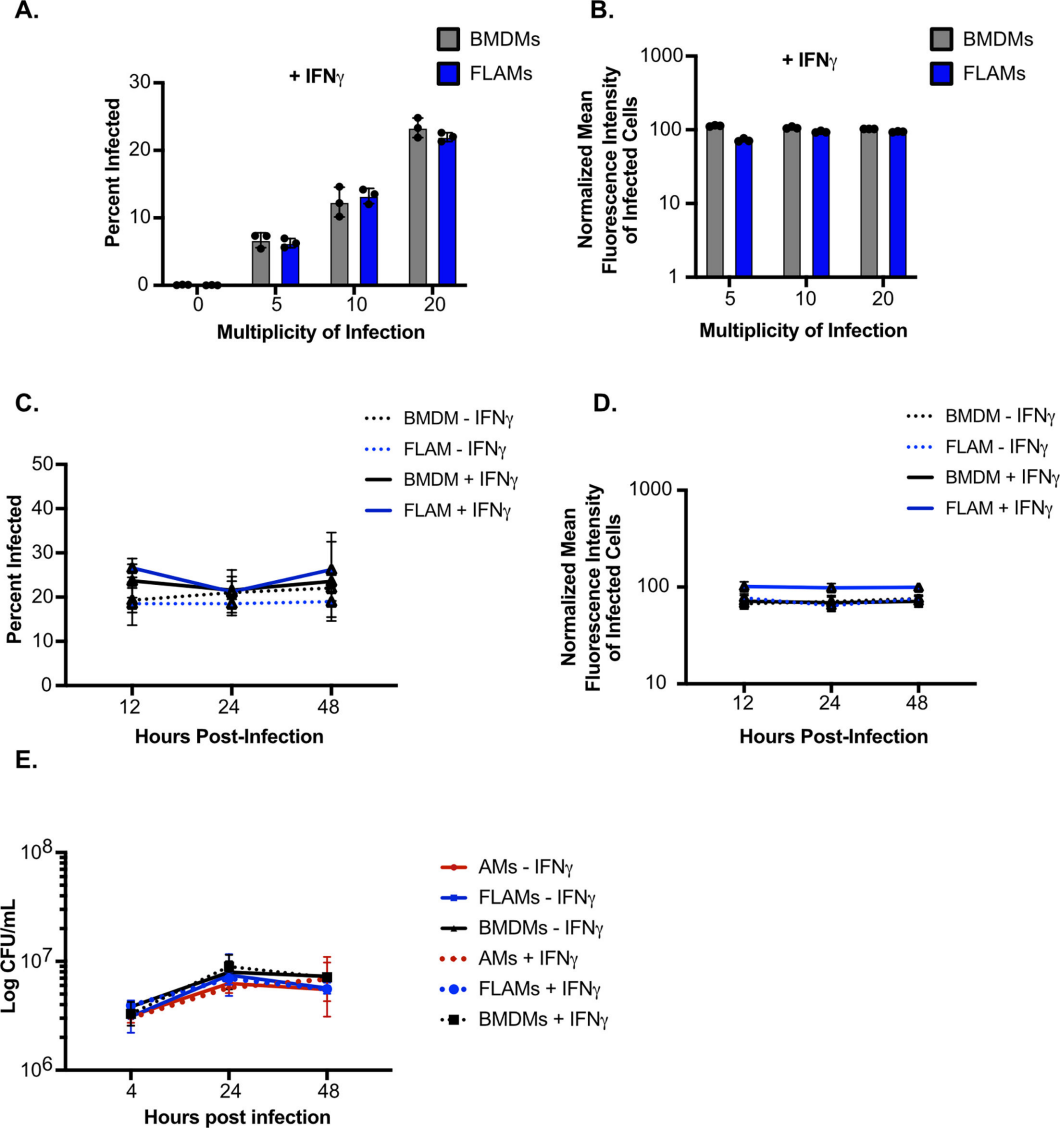
IFNγ activation of BMDMs AMs, or FLAMs does not alter Mab uptake or intracellular control. (**A**) The percent of infected cells and the (**B**) MFI of infected cells were quantified by flow cytometry from IFNγ-activated (25 ng/mL) BMDMs or FLAMs that were infected with mEmerald-Mab at the indicated MOIs for 4 h. (**C**) The percent of infected cells and the (**D**) MFI of infected cells were quantified by flow cytometry from untreated or IFNγ-activated (25 ng/mL) BMDMs or FLAMs that were infected with mEmerald-Mab (MOI 5) for the indicated time points post-infection. (**E**) Viable intracellular Mab was quantified by CFU assay from untreated or IFNγ-activated (25 ng/mL) BMDMs, FLAMs, or AMs that were infected with mEmerald-Mab (MOI 5) for the indicated time points post-infection. Shown data are representative of three independent experiments with three replicates per group. Untreated data are from the same experiment shown in Fig. 1D through F, and appropriate multiple hypothesis testing corrections have been made. No significance was observed in panels **A** and **B**. For panels **C** and **D**, no significance was observed between IFNγ-activated BMDMs and FLAMs or between resting and activated BMDMs or FLAMs. For panel **E**, no significance was observed between IFNγ-activated BMDMs, FLAMs and AMs or between resting and activated BMDMs, FLAMs and AMs.

Patients who develop chronic Mab respiratory infections are characterized by previous lung damage and ongoing respiratory dysfunctions (9, 45). In these patients, the baseline inflammatory state of macrophages is different from that of resting macrophages. IFNγ is produced during inflammatory responses and is a key cytokine that activates macrophages and can drive restriction of intracellular pathogens (26, 27). We previously showed that the IFNγ response of FLAMs is distinct from that of BMDMs, but how this alters the host response to infection remains unknown(19). To test if IFNγ differentially alters Mab-host interaction in FLAMs and BMDMs, cells were activated overnight with IFNγ and then infected with mEmerald-Mab before multiple parameters were analyzed. First, we examined whether IFNγ altered Mab uptake by BMDMs and FLAMs. We found that 4 h following infection, IFNγ did not alter uptake efficiency between BMDMs and FLAMs at increasing MOIs (Fig. 5A and B). We next examined intracellular dynamics over time. Using flow cytometry, we noted a slight increase in the percentage of infected cells in both BMDMs and FLAMs, but this remained stable through 48 h of infection (Fig. 5C). We also noted a similar MFI of infected cells between resting and IFNγ-activated BMDMs and FLAMs (Fig. 5D). To confirm these results, this experiment was repeated, and primary AMs from bronchial lavage fluid were included. Cells were infected and then were lysed over time up to 48 h following infection, followed by quantification of colony-forming units (CFU) (Fig. 5E). In line with our flow cytometry approaches, we found no significant differences in Mab viability between resting or IFNγ-activated AMs, FLAMs, or BMDMs (Fig. 5E). These data suggest that IFNγ has a limited antimicrobial role in controlling intracellular Mab, which persist in activated AMs, FLAMs, and BMDMs.

### IFNγ drives more cell death and inflammatory cytokines following Mab infection in both BMDMs and FLAMs

**Fig 6:**
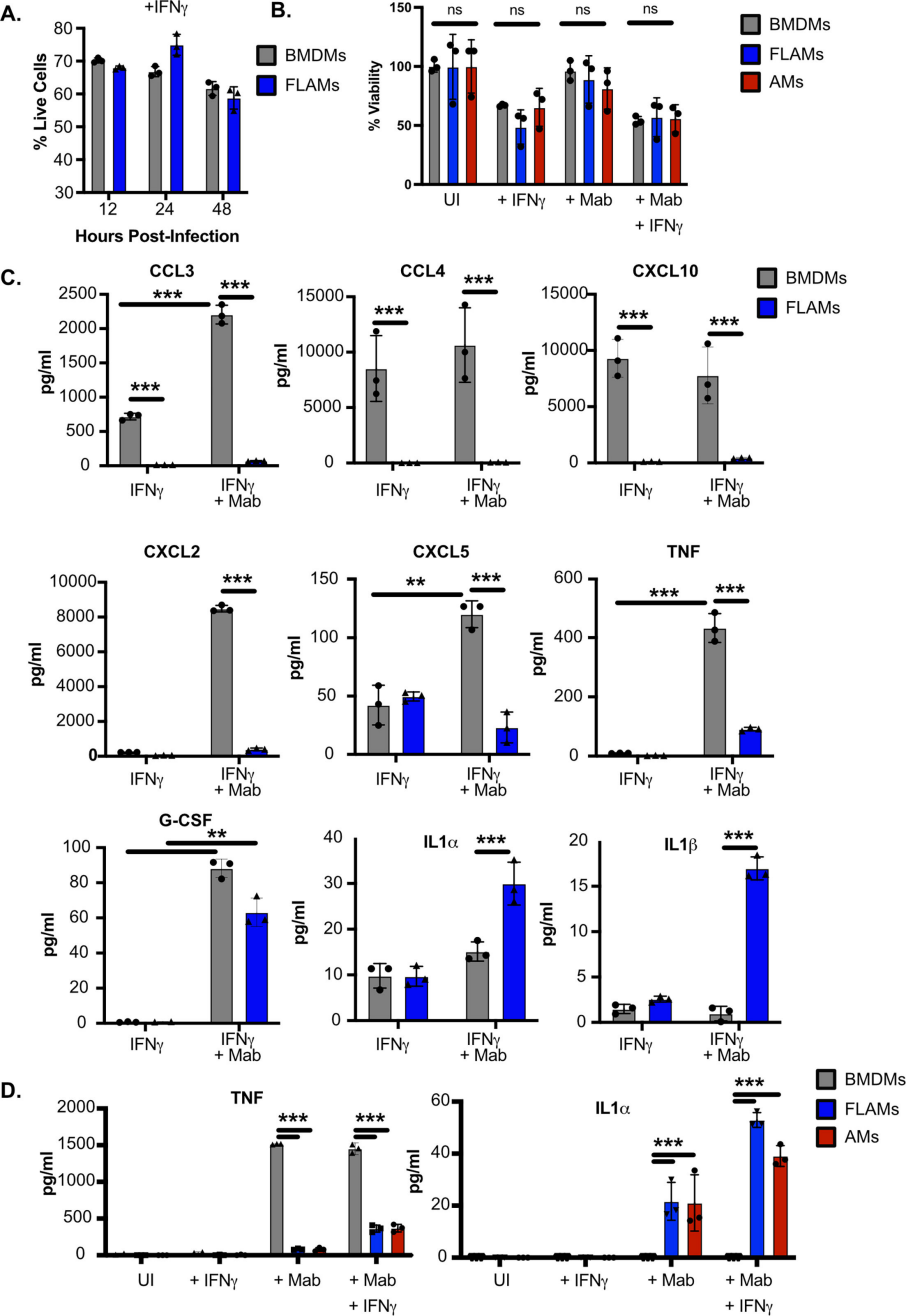
The inflammatory response of IFNγ-activated BMDMs remains distinct compared to those of AMs and FLAMs. (**A**) Shown is the percent of viable cells (Live/Dead stain negative) quantified by flow cytometry for IFNγ-activated (25 ng/mL) BMDMs or FLAMs infected with mEmerald-Mab (MOI 5) for the indicated time point. (**B**) The percent of viable cells 24 h following mEmerald-Mab infection of untreated or IFNγ-activated (25 ng/mL) BMDMs, FLAMs, or primary AMs (MOI 5) using CellΤiter-Glo. The percent cells that were alive was determined by normalizing each sample to the mean of the uninfected cell type control. (**C**) Shown is the concentration of cytokines from the supernatants of IFNγ-activated (25 ng/mL) BMDMs or FLAMs infected with mEmerald-Mab (MOI 5) 24 h following infection. (**D**) Resting or IFNγ-activated (25 ng/mL) BMDMs, FLAMs, or primary AMs were infected with mEmerald-Mab (MOI 5), and 24 h later, TNF and IL-1α were quantified in the supernatants by ELISA. Panels** A, B, and D** are representative of three independent experiments with three biological replicates per group. Panel **C **is from a single multiplex experiment with three biological replicates per group. ns: not significant, **P < 0.01, ***P < 0.001 by two-way ANOVA with Tukey’s correction for multiple comparisons.

We next examined how IFNγ alters the host response to Mab infection. IFNγ-activated BMDMs and FLAMs were infected with mEmerald Mab, and changes in cell viability were quantified over time using two orthologous approaches. First, cell viability of infected cells was quantified using flow cytometry 12, 24, and 48 h post-infection (Fig. 6A). Our results showed significant cell death in both BMDMs and FLAMs over time but no differences between cell types. To confirm these results, we infected primary AMs in addition to FLAMs and BMDMs and then quantified cell death the following day. We found no significant differences in cell death between any cell type, although IFNg increased cell death independently of infection (Fig. 6B). These data suggest that while IFNγ activation increases cell death pathways in both BMDMs and FLAMs, it does so independently of Mab infection.

We next dissected if the inflammatory response of BMDMs and FLAMs was altered by IFNγ activation. IFNγ-stimulated FLAMs or BMDMs were left uninfected or were infected with Mab at an MOI of 5, and 48 h later, pro-inflammatory cytokines in the supernatants were quantified by a multiplex Luminex assay (Fig. 6C). We observed that IFNγ activation alone drove a higher baseline expression of CCL3, CCL4, and CXCL10 in BMDMs, but only CCL3 further increased an additional 4-fold following Mab infection. In contrast, IFNγ-activated FLAMs did not secrete these cytokines. Similar to resting cells, IFNγ-activated BMDMs induced high levels of CXCL2 and CXCL5 following Mab infection, but this was not observed in FLAMs. Notably, G-CSF was similarly induced in both IFNγ-activated Mab-infected BMDMs and FLAMs. We observed that TNF was induced in IFNγ-activated Mab-infected FLAMs, yet the overall TNF levels remained significantly lower than those in BMDMs. Finally, we found that while both IL-1α and IL-1β were significantly induced in IFNγ-activated infected FLAMs but not in BMDMs, the amount of these cytokines secreted was generally low. To validate these results, we infected IFNγ-activated primary AMs in addition to FLAMs and BMDMs and then quantified TNF and IL-1α secretion the following day (Fig. 6D). Both AMs and FLAMs secreted significantly lower levels of TNF compared to BMDMs but significantly more IL1α. Together, these results show that while IFNγ alters the inflammatory response of both BMDMs and FLAMs, these cell types continue to drive distinct cytokine profiles following Mab infection.

### IFNγ-activation drives distinct transcriptional changes in Mab-infected FLAMs and BMDMs

**Fig 7:**
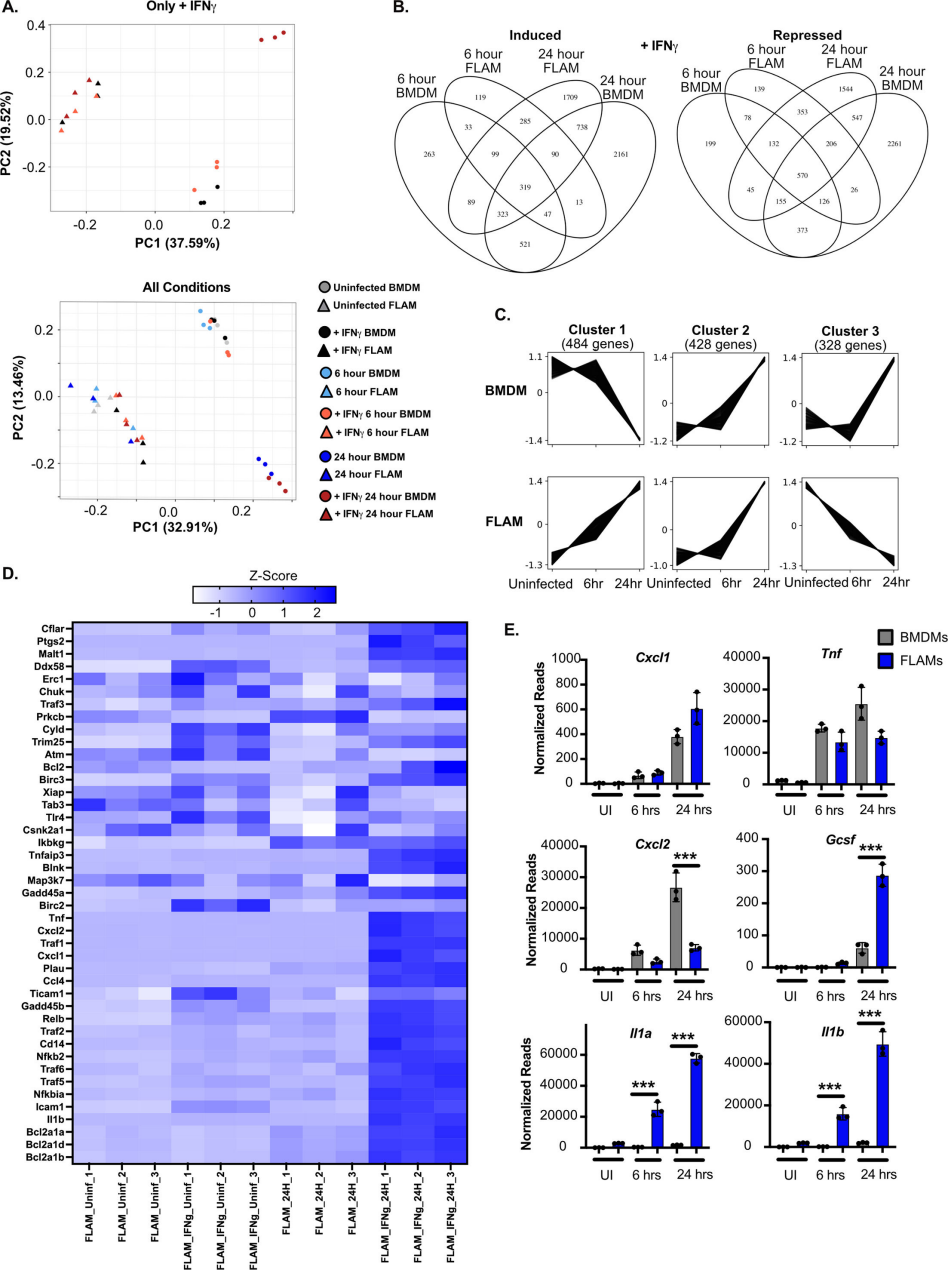
IFNγ drives more robust NF-κΒ activation in Mab-infected FLAMs. (**A**) A principal component analysis (PCA) plot comparing the similarity of the transcriptional responses of (Top) IFNγ-activated BMDMs and FLAMs during Mab infection and (Bottom) all conditions examined in BMDMs and FLAMs during Mab infection. (**B**) Venn diagrams showing shared and unique genes that were significantly induced or repressed in IFNγ-activated BMDMs and FLAMs during Mab infection using DeSeq2. (**C**) Representative clusters of genes that respond similarly to Mab infection in IFNγ-activated BMDMs and FLAMs over time. (**D**) Normalized counts of NF-κΒ genes from the KEGG pathway set were compared across the indicated conditions in FLAMs and are expressed as a heatmap. The color scale represents the z-score calculated from normalized read counts across samples for each gene. (**E**) Normalized counts of a subset of NF-κΒ genes that were differentially regulated in BMDMs (gray) and FLAMs (blue) during Mab infection. ***P < 0.001 based on adjusted *P*-values using DESeq2 comparisons.

We next compared the global transcriptomic changes of IFNγ-activated FLAMs and BMDMs during Mab infection. BMDMs and FLAMs were activated with IFNγ overnight and then infected with Mab at an MOI of 5. RNA was isolated from cells 6 and 24 h later, followed by RNA-sequencing analysis (Table S1). To identify general differences in the transcriptomes, we visualized the data using PCA (Fig. 7A). When we examined only IFNγ-activated conditions with BMDMs and FLAMs, we noted a separation between cell types along the PC1 axis. We also noted separation on the PC2 axis for BMDMs infected with Mab for 24 h. In contrast, we did not observe any significant movement along PC2 with FLAMs. This analysis was remarkably similar to our PCA from resting BMDMs and FLAMs (see Fig. 3A). To directly compare resting and IFNγ-activated cells, we conducted a new PCA from all conditions (Fig. 7A). While FLAMs clustered together for all conditions, both resting and IFNγ-activated BMDMs infected with Mab for 24 h showed a strong shift from the other BMDM conditions. These data suggest that Mab infection drives a more robust transcriptional change in BMDMs compared to FLAMs.

We next compared genes that were differentially expressed in either IFNγ-activated FLAMs or BMDMs over time following Mab infection (Fig. 7B). Similar to resting cells, we noted almost two times the number of unique induced and repressed genes in BMDMs at both 6 h and 24 h post-infection compared to FLAMs. When we compared the lists of differentially expressed genes from IFNγ-activated and resting cells from the same condition, we found that IFNγ activation drives more changes to gene expression following Mab infection than Mab infection alone in both FLAMs and BMDMs. To identify key patterns of transcriptional changes during Mab infection of BMDMs and FLAMs, we used clustering analysis to identify groups of genes whose expression changes similarly across each IFNγ-activated condition (Table S2). We found four clusters of genes, and using pathway and transcription factor analysis, we identified transcriptional networks that were associated with each cluster (Fig. 7C). Interestingly, cluster 1 contains genes that are induced in IFNγ-activated Mab-infected FLAMs but are repressed in BMDMs. Among the top enriched KEGG pathways in this cluster was oxidative phosphorylation, suggesting key differences in the metabolic response of IFNγ-activated BMDMs and FLAMs to Mab infection. We also observed that Cluster 3 contained genes that were induced in IFNγ-activated Mab-infected BMDMs, but not FLAMs. In contrast to resting cells, we did not observe a significant enrichment of the NF-κΒ pathway from this cluster. This suggests that IFNγ activation of FLAMs drives a more robust NF-κB response following Mab infection. To examine this directly, we compared the normalized reads from FLAMs for NF-κB-dependent genes examined in Fig. 3 above. We found that IFNγ activation of FLAMs drove significantly higher expression of NF-κB-dependent genes following Mab infection (Fig. 7D). Thus, NF-κΒ can be more robustly activated in Mab-infected FLAMs following IFNγ stimulation. When we examined a subset of genes examined by our multiplex analysis, we saw strong agreement for the majority of cytokines, including *Cxcl1*, *Cxcl2*, *Tnf,* and *Gcsf* (Fig. 7E). Interestingly, while we also observed that only FLAMs induce *Il1a* and *Il1b* in response to Mab, the magnitude of these changes at the transcriptional level was significantly higher compared to that of the released cytokines.

### The lack of *Nos2* expression following Mab infection of IFNγ-activated FLAMs is overcome by the stabilization of HIF1α

**Fig 8:**
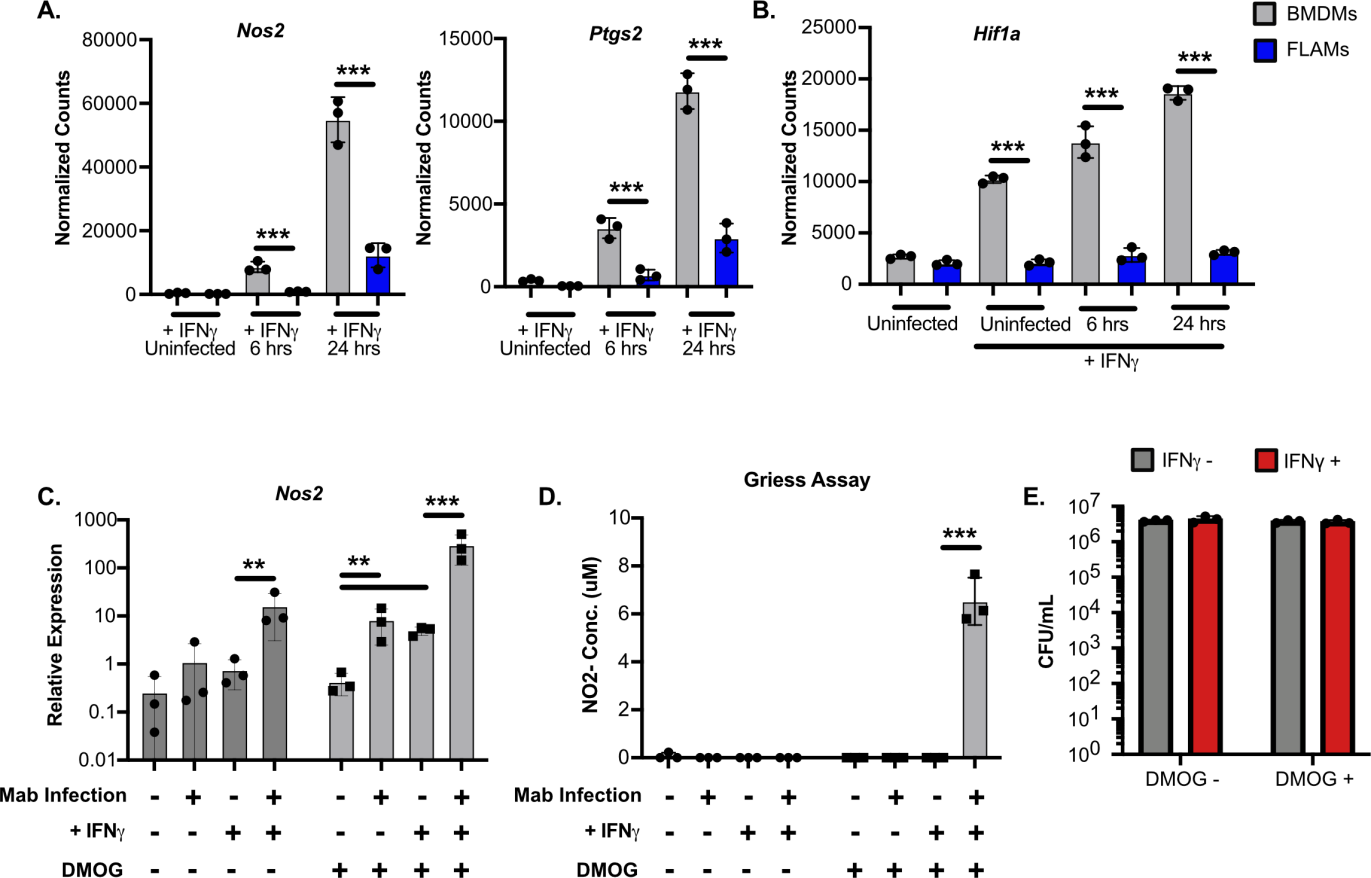
Chemical activation of HIF1α increases Nos2 expression in IFNγ-activated FLAMs following Mab infection. (**A**) Normalized counts of *Nos2* and *Ptgs2* in IFNγ-activated BMDMs (gray) and FLAMs (blue) during Mab infection. (**B**) Normalized counts of *Hif1a* in resting or IFNγ-activated BMDMs (gray) and FLAMs (blue) during Mab infection from the RNAseq dataset. (**C**) FLAMs were left untreated or were treated with 25 ng/mL of IFNγ and/or 250 μM DMOG, and 12 h later, cells were infected with Mab for 4 h. Twenty-four hours later, RNA was isolated, and qRT-PCR was used to determine the relative expression of *Nos2* compared to *Bact* controls. (**D**) FLAMs were left untreated or were treated with 25 ng/mL of IFNγ and/or 250 μM DMOG, and 12 h later, cells were infected with Mab for 4 h. Twenty-four hours later, 50 μL of cell supernatants was used to measure the concentration of NO_2_^−^ in each condition. (**E**) CFU enumeration of Mab-infected FLAMs with and without DMOG at 24 h. Statistical significance for normalized reads was determined based on adjusted *P*-values using DeSeq2. **P < 0.01; ***P < 0.001 by two-way ANOVA with Tukey’s correction for multiple comparisons.

An important role of IFNγ activation is to induce antimicrobial compounds, such as nitric oxide (NO). NO is known to play an important role during mycobacterial infections, yet whether it is differentially regulated during Mab infection remains unclear (22, 46–48). When we compared gene sets between FLAMs and BMDMs during IFNγ activation, we noted significant differences in the expression of *Nos2* and *Ptgs2* (Fig. 8A). Previous studies found these genes are induced in both a HIF1α-dependent manner that is also associated with a metabolic shift to aerobic glycolysis (49–51). Given that we observed a specific increase in oxidative phosphorylation pathways in IFNγ-activated FLAMs, we hypothesized that HIF1α may not be as robustly induced in FLAMs compared to BMDMs. When we examined the expression of *Hif1a* in our RNA-seq data set, we observed that while *Hif1a* expression was significantly induced during Mab infection of IFNγ-activated BMDMs, the expression remained unchanged in FLAMs (Fig. 8B). We next wanted to test whether the activation of HIF1α was sufficient to increase *Nos2* expression in FLAMs. FLAMs were left resting or were activated with IFNγ in the presence or absence of the HIF1α stabilizer, dimethyloxalylglycine (DMOG). Cells were infected with Mab, and 24 h later, RNA was isolated, and the expression of *Nos2* was quantified by RT-PCR. We found that IFNγ-activated Mab-infected FLAMs treated with DMOG induced 10 times more *Nos2* than vehicle control-treated cells (Fig. 8C). When we repeated this experiment and examined nitrite production using a Griess assay, we observed a similar result (Fig. 8D). FLAMs only produced detectable levels of nitrite in the presence of DMOG following IFNγ activation and Mab infection. Importantly, the addition of DMOG did not change Mab viability (Fig. 8E). These data suggest that HIF1α is not robustly induced in IFNγ-activated FLAMs during Mab infection, and this results in changes to the inflammatory response, including low induction of *Nos2* and NO production. Thus, IFNγ-activated FLAMs activate distinct pathways following Mab infection compared to BMDMs.

## DISCUSSION

Respiratory infections with Mab are an increasing clinical concern. Given the high failure rates of antibiotic therapy and no protective host-directed therapies or vaccines available, understanding how Mab is effectively controlled in some hosts and not in others is critically important. Here, we leveraged new *ex vivo* models of lung-specific alveolar macrophages to better understand key differences in the innate immune response during Mab infection between distinct macrophage subtypes. Our data suggest that while BMDMs, AMs, and FLAMs control Mab infection independently of IFNγ activation, they sense and respond to Mab infection in distinct ways. Myeloid-derived BMDMs were more inflammatory, driving higher levels of cytokines, chemokines, and activation markers; in contrast, primary AMs and FLAMs were less inflammatory, with lower expression of inflammatory genes and cytokines. Our findings highlight the importance of defining host-pathogen interactions in a range of tissue-relevant immune cells to better identify mechanisms that may contribute to control of Mab and other respiratory infections.

Mab infects macrophages, which serve as a key intracellular niche throughout infection (18, 52, 53). The intracellular dynamics of Mab, however, remain relatively uncharacterized. In contrast to other studies, our findings using an array of readouts, including bulk CFU counts as well as single-cell flow cytometry approaches, suggest that Mab does not replicate to high levels in either BMDMs or FLAMs. Instead, intracellular Mab is relatively steady over the first 2 days of infection while not inducing high levels of cell death. Whether this steady state is indicative of static growth or equivalent rates of growth and death remains to be determined but will be important to better understand the mechanisms controlling early Mab interactions with macrophages. Given the importance of IFNγ in controlling mycobacterial infections and the inflammatory environment seen in patients susceptible to Mab infection, it was surprising that we found very little effect of IFNγ activation on intracellular dynamics of Mab infection. This is in contrast to *Mycobacterium tuberculosis* (Mtb)*,* which is readily restricted in IFNγ-activated BMDMs. It is possible that Mab is more resistant than Mtb to intracellular antimicrobial poisons. Alternatively, Mab may be maintained in a distinct intracellular compartment that is resistant to IFNγ-inducible effectors. Future work will be needed to define the intracellular dynamics of Mab infections, with particular focus on important macrophage pathways that control the replication of this opportunistic pathogen.

A key finding throughout our study was the distinct inflammatory responses that Mab activated in FLAMs and BMDMs. In general, BMDMs were more hyper-inflammatory, producing higher levels of chemokines like CXCL1 and CXCL2 and cytokines like TNF and G-CSF in both the presence and absence of IFNγ activation. In contrast, AMs and FLAMs produced IL-1a while BMDMs did not. Yet, what regulates the cell type specificity of IL-1α is unknown. Further work will need to examine if the differential expression of lipid metabolism and peroxisomes drives expression and if caspase 1 or other proteases contribute to the maturation. One mechanism driving more inflammation in BMDMs than AMs is the activity of transcription factors, such as Nrf2 and NF-κB. Our data suggest that while loss of Nrf2 results in increased basal inflammation, it does not alter Mab-dependent inflammatory responses in FLAMs. In contrast, we found that NF-κB was driving high expression of a subset of cytokines in BMDMs, including TNF and CXCL10, yet NF-κB was not robustly induced in FLAMs. While IFNγ activation drove more NF-κB activation in FLAMs, many key genes remained lower than in BMDMs. This included *Nos2*, which we found could be increased in FLAMs treated with IFNγ and the HIF1α activator DMOG. HIF1α is known to be interlinked with the shift of IFNγ-activated cells toward aerobic glycolysis (50, 51). Thus, taken together, we speculate that one key driver of the distinct activation of NF-κΒ and HIF1α in FLAMs and BMDMs during Mab infection is metabolic differences. Future studies will need to directly examine the dynamics of HIF1a expression, protein stability, and localization. Understanding how metabolism drives differences between FLAMs and BMDMs and manipulating these pathways genetically or chemically in resting, activated, and infected conditions will be an important next step to define the underlying mechanisms of inflammatory regulation in distinct macrophage subsets.

Mab respiratory infections are cleared by the majority of immunocompetent patients, yet the mechanisms mediating this control remain unclear. AMs are the first line of immune defense, and our results using FLAMs as a model for AMs suggest that these cells control Mab infection without driving a severe inflammatory response. Patients who are susceptible to Mab have preexisting lung conditions that disrupt the normal state of alveolar macrophages through activation, including IFNγ stimulation, and the recruitment of myeloid-derived macrophages. The inflammatory environment in the lungs and the ontogeny of airway macrophages during Mab infection may further contribute to inflammation, driving more tissue damage and increasing the severity of respiratory disease. Continuing to dissect how ongoing inflammation in susceptible patients dampens pathogen control without driving lung disease will aid in the identification of new therapeutic opportunities that could be used synergistically with antibiotic therapy.

One important shortcoming of our study is that the Mab laboratory strain we used (ATCC19977) is a smooth colony variant. It is well known that Mab isolates can present as smooth variants through the expression of GPLs, with the loss of these GPLs resulting in rough colony variants (53–55). In clinical studies, both smooth and rough variants are isolated from patients, with data suggesting the host environment drives increased transition from the smooth to rough variant during infection (53, 56). Rough Mab drives more inflammatory cytokines, such as TNF, compared to smooth Mab when infecting BMDMs (52, 57). Rough isolates are also suggested to result in bacterial cording phenotypes that further drive inflammatory responses (35, 54). Whether other bacterial factors, in addition to GPL expression, drive inflammation in macrophages remains to be fully understood. Furthermore, while FLAMs are proving to be a useful *ex vivo* model for AMs, whether these responses hold up within the pulmonary environment will need to be confirmed in future work.

Taken together, our study identified many key differences in the inflammatory response of distinct macrophage subsets following infection with Mab. These differences in the innate immune response occurred independently of differences in bacterial control or cell death. Thus, the innate immune wiring of distinct macrophage subsets is unique. These results can now be used to identify the importance of distinct innate sensing pathways during infection with Mab and other respiratory pathogens to develop new host-directed therapies that prevent infection while limiting inflammatory damage to the lungs.

## MATERIALS AND METHODS

### Mab culture conditions

mEmerald GFP-expressing Mab (ATCC 19977) strains were generated as previously described (30). All mEmerald GFP-expressing Mab cultures were grown aerobically at 37°C in Middlebrook 7H9 medium supplemented with 1% glycerol, 0.05% Tween-80, and 10% Middlebrook OADC (oleic acid, dextrose, catalase, and bovine albumin). To select for mEmerald GFP-expressing Mab, zeocin (Invivogen) was included at a final concentration of 5 μg/mL.

### Animal experiments

All cell isolation procedures involving live mice were performed in accordance with the recommendations from the Guide for the Care and Use of Laboratory Animals of the National Institutes of Health and the Office of Laboratory Animal Welfare. Mouse studies were performed using protocols approved by the Institutional Animal Care and Use Committee (IACUC). All mice were housed and bred under specific pathogen-free conditions and in accordance with Michigan State University (PROTO202200127) IACUC guidelines. All mice were monitored and weighed regularly. C57BL6/J mice (# 000664) and Nrf2^−/−^ mice (# 017009) were purchased from The Jackson Laboratory.

### BMDM, AM, and FLAM cell isolation, maintenance, and culture conditions

Primary BMDMs were generated by isolating marrow from femurs and plating in RPMI containing 25 ng/mL M-CSF (R&D Systems) and 10% FBS for 8–10 days. Cells were then washed with PBS and lifted with PBS-EDTA prior to plating for experiments. In indicated experiments, HoxB8-conditionally immortalized macrophages were used. These cells were isolated from C57BL6/J mice and were maintained in media containing 30 ng/mL recombinant mGM-CSF (R&D Systems), 10% fetal bovine serum (FBS), and 0.5μM β-estradiol, as previously described (58–60). To generate BMDMs, cells were washed in PBS to remove estradiol and then plated in RPMI containing 25 ng/mL M-CSF (R&D Systems) and 10% FBS. Then, 5 to 7 days later, cells were plated for experiments as described in figure legends. For FLAMs, C57BL6/J pregnant dam mice were euthanized by CO_2_ prior to cervical dislocation, and fetal liver-derived cells were obtained as previously described (36). Cells were cultured in RPMI (ThermoFisher) containing 10% FBS, 30 ng/mL recombinant mGM-CSF (R&D Systems), and 20 ng/mL recombinant hTGF-β1 (R&D Systems). For AMs, C57BL6/J mice were euthanized by CO_2_ before exsanguination, and bronchoalveolar lavage fluid was collected by flushing the lungs with warm PBS-EDTA (10 mM), as done previously (41). Cells were pelleted by centrifugation at 500 × *g* and washed with PBS and then resuspended and cultured in RPMI (ThermoFisher) containing 10% FBS, 30 ng/mL recombinant mGM-CSF (R&D Systems), and 20 ng/mL recombinant hTGF-β1 (R&D Systems). All macrophages were incubated in 5% CO_2_ at 37°C. FLAMs were monitored regularly by flow cytometry for the continued expression of alveolar macrophage markers including high SiglecF and low CD14.

### Macrophage infections and assays

#### Mab infection of macrophage monolayers

Single-cell Mab suspensions were prepared by resuspending logarithmic phase bacteria in the appropriate macrophage cell culture media, followed by a soft spin at 58 × *g* to pellet large bacterial clumps. Macrophages were seeded at 5 × 10^5^/well in a 12-well plate, and single-cell Mab supernatants were used for macrophage infection at the indicated MOIs. To ensure a synchronized infection, plates were centrifuged at 58 × *g* for 5 min. Four hours later, the infection medium was removed, and the medium containing 64μg/mL amikacin was added for the remainder of the experiment. At each indicated time point (4 to 48 h), macrophages were washed with PBS, lifted from plates by scraping or using Accutase (BioLegend) treatment, and then fixed in 4% paraformaldehyde. Infected macrophages were quantified using the BD LSR II (BD Biosciences) or Attune CytPix Flow Cytometer (Thermo Fisher Scientific) at the Michigan State University Flow Cytometry Core. Live and single macrophages were identified using forward and side scatter and fixable live dead dyes (BioLegend), and the mean fluorescent intensity of infected cells was determined by the fluorescence in the GFP channel. All experiments included uninfected and unstained controls to set gates for infected macrophage quantification. Analysis was performed using FlowJo V10.

#### Mab infection of IFNγ activated macrophages

For IFNγ activated macrophage experiments, cells were treated with 25 ng/mL IFNγ (R&D Systems) for 16–20 h. Following macrophage activation, the IFNγ-containing medium was removed, and infection medium was added as described above. Four hours later, infection medium was removed, and medium containing 64 μg/mL amikacin and 25 ng/mL IFNγ was added for the remainder of the experiment.

#### Quantification of Mab intracellular growth

For intracellular growth experiments, macrophages were lysed at the indicated time points (12, 24, and 48 h) with sterile cold distilled water. Following 10-fold serial dilutions on Middlebrook 7H10 agar supplemented with Middlebrook OADC and 5 μg/mL zeocin, samples were plated to perform colony-forming unit (CFU) counts. Colonies were enumerated after 4–5 days of incubation at 37°C.

#### Quantification of cell death

For flow cytometry cell death experiments, macrophages were lifted at the indicated time points (4 to 48 h) and then stained with Zombie Red Live/Dead stain (Biolegend). Once stained, cells were washed with PBS and fixed in 4% paraformaldehyde before being analyzed by flow cytometry. For CellTiter Glo (Promega), cells were seeded in 96-well opaque plates the day prior to the experiment and stimulated with 25 ng/mL of IFNγ (R&D Systems) overnight. The following day, macrophages were infected as described above. At the indicated time points, viability measurements were performed following CellTiter Glo kit instructions. Briefly, 100 μL of CellTiter Glo reagent was added directly to each well, and plates were incubated for 2 min with shaking to induce cell lysis. Plates were then incubated for another 10 min at room temperature to stabilize the luminescent signal, and luminescence was measured on a Spark multimode microplate reader (Tecan). Luminescence signal was normalized to uninfected cells for each condition.

#### Inhibitor/activator experiments

For experiments blocking NF-κB, the inhibitor BAY 11-7082 (Invivogen) was resuspended in ethanol and diluted in media to a final concentration of 5 μM for experiments. For experiments activating HIF-1α, DMOG (Sigma-Aldrich) was resuspended in DMSO and diluted to a final concentration of 250 μM in media.

#### Immunofluorescence microscopy

BMDMs and FLAMs were seeded in a black, glass-bottom 96-well plate (Cellvis # P96-1.5H-N) coated with poly-D-lysine at a density of 70,000/well and were allowed to adhere for 24 h. Cells were stimulated with LPS or infected with mEmerald-Mab ATCC19977 at an MOI of 5. After 6 h post-infection, cells were washed with PBS and fixed with 4% paraformaldehyde. Immunostaining was performed by first blocking the specimen with 5% BSA and 0.3% Triton X-100 for 1 h. The anti-NFkB primary antibody was diluted and incubated overnight at 4°C (NFkBp65 D14E12 Rabbit mAb 1:1000, Cell Signaling Technology # 8242). The next day, cells were incubated with fluorochrome-conjugated secondary antibody Alexa Flour 633 Goat anti-Rabbit IgG for 1 h in the dark (1:1,000, Invitrogen # A21070). Finally, DAPI staining was performed to localize the nuclei of the cells. Cells were washed with PBS after the last staining, and 200 μ L of PBS was used as mounting media. Cells were imaged using CellVoyager CQ1 Benchtop High-Content Analysis System (Yokogawa) or the Leica MICA confocal microscope (Leica Microsystems) with 20× HC PL APO CS2 and 63× HC PL APO CS2 objectives). Images were analyzed using the imaging analysis software CellProfiler v4.2.6 (Script available at Olive Lab Github: https://github.com/SoleSove/OliveLab_MabCellProfiler). ImageJ was used to adjust presentation and add in scale bars for each figure.

### Griess assay

The quantity of nitric oxide produced by macrophages was determined by measuring its stable end product nitrite (Griess Assay, Promega). Briefly, 50 uL of the supernatant was transferred to a 96-well plate, followed by 50 μL of sulfanilamide and 50 μL of N-1-napthylethylenediamine dihydrochloride (NED) under acidic conditions. Following incubation, absorbance was measured at 540 nm on a Tecan Spark 20 M plate reader, and nitrite concentrations were calculated using a standard nitrite curve as per the manufacturer’s instructions.

### RNA isolation and quantitative RT-PCR

For the RNA-seq experiments, 5 × 10^6^ cells were plated and treated with IFNγ for 18 h prior to infection. The following day, the medium was removed, and the infection medium without IFNγ was added in a volume of 2 mL. Six hours later, the medium was removed and replaced with fresh medium for an additional 18 h, or cells were lysed directly in 1,000 μL of TRIzol reagent (Life Technologies) and incubated for 5 min at room temperature. At 24 h following infection, the remaining cells were lysed in 1,000 μL of TRIzol reagent. To purify RNA, 200 μL of chloroform was added to the cell homogenates, vortexed, and centrifuged at 10,000 × *g* for 18 min at 4°C to separate nucleic acids. The upper aqueous phase was removed and combined with equal parts ethanol. This mixture was placed into a collection tube, and protocols provided by the Direct-zol RNA extraction kit (Zymo Research) were followed. Quantity and purity of the RNA were checked using a Qubit (LifeTechnologies), and it was diluted to 50 ng/mL in nuclease-free water for RNAsequencing or 5 ng/mL for RT-PCR. For RT-PCR, the One-Step SYBR Green RT-PCR kit (Qiagen) reagents were used to amplify RNA according to the manufacturer’s instructions. Amplifications were monitored using the QuantStudio3 (ThermoFisher). Relative mRNA expression levels were calculated after normalization to β-actin using the primers given below: *Nos2* Forward: 5′ GTT CTC AGC CCA ACA ATA CAA GA 3' *Nos2* Reverse: 5′ GTG GAC GGG TCG ATG TCA C 3′ *Bactin* Forward 5′ GGC TGT ATT CCC CTC CAT CG 3′ *Bactin* Reverse 5′ CCA GTT GGT AAC AAT GCC ATG T 3′.

### Cytokine analysis

Where indicated, supernatants were filter-sterilized through a 0.2-micron filter before cytokines were quantified by a Luminex multiplex assay (Eve Technology). In addition, filter-sterilized supernatants from Mab-challenged and control macrophages were harvested, and the indicated cytokine protein levels were determined using CXCL10, TNF, IL6, or IL-1α DuoSet ELISA kits (R&D Systems) following the manufacturer’s instructions. Absorbance (450 nm) was detected on a Spark multimode microplate reader (Tecan).

### RNA sequencing and analysis

The Illumina Stranded mRNA Library Prep kit (Illumina, Cat no. 20040534) with IDT for Illumina RNA Unique Dual Index adapters was used for library preparation following the manufacturer’s recommendations but using half-volume reactions. Qubit dsDNA HS (ThermoFischer Scientific, Cat no. Q32851) and Agilent 4200 TapeStation HS DNA1000 assays (Agilent, Cat no. 5067–5584) were used to measure the quality and quantity of the generated libraries, respectively. The libraries were pooled in equimolar amounts, and the Invitrogen Collibri Quantification qPCR kit (Invitrogen, Cat no. A38524100) was used to quantify the pooled library. The pool was loaded onto two lanes of a NovaSeq S4 flow cell at Genewiz Sequencing, and sequencing was performed in a 2 × 150 bp paired-end format using a NovaSeq 6000 v1.5 100-cycle reagent kit (Illumina, Cat no. 20028316). Base-calling was performed with Illumina Real-Time Analysis (RTA; v3.4.4), and the output of RTA was demultiplexed and converted to the FastQ format with Illumina Bcl2fastq (v2.20.0).

This work was supported in part by Michigan State University through computational resources provided by the Institute for Cyber-Enabled Research. RNA-seq read quality assessment, mapping, and counting were performed using a custom pipeline built in Snakemake v7.32.4 (https://github.com/kaylaconner/olivelab-rnaseq/tree/main) (61). Read quality was assessed using FastQC version 0.12.1 (62). Read mapping was performed against the GRCm39 mouse reference genome using Bowtie2 v2.5.1 (63). Aligned read counts were assessed using the featureCounts function from the Subread package v2.0.6 (64). Differential gene expression analysis was conducted using the DESeq2 package v1.42.0 in R v4.3.2 (65). Pre-filtering was performed to keep only genes that had > 10 counts in three or more samples.

Principal component analyses (PCA) were performed using the prcomp function with scaling on normalized count matrices of all genes in R v4.3.2, and PCA visualization was done using the autoplot function in ggplot2 v3.4.4 (66). Magnitude/amplitude (MA) plots were produced by plotting Log2(Fold Change) and Log2(Base Mean) values from DESeq2 in ggplot2 v3.4.4 (66). Heatmaps were produced in GraphPad Prism v9.4.1 using normalized counts data from DESeq2. Venn diagrams were produced using the VennDiagram package n v1.7.3 in R v4.3.2 (67). Gene clustering analysis was performed using Clust v1.18.0 (68) (three replicates per condition with automatic normalization; -t 0.2 for Fig. 3C; -t 2.0 for Fig. 7C). All Gene Ontology analyses were performed using the g:OSt functional profiling tool from the g:Profiler web server version *e111_eg58_p18_30541362* (database last updated on 25 January 2024) (69). All raw sequencing data, including analyzed normalized counts, are available at the Gene Expression Omnibus (GEO), accession number GSE264083.

### Statistical analysis and data visualization

Statistical analysis was performed using Prism Version 10 (GraphPad), as indicated in the figure legends. Data are presented, unless otherwise indicated, as mean ± standard deviation. One-way or two-way ANOVA followed by Tukey’s *post ho*c test was used to identify significant differences between multiple groups, and Student’s *t*-tests were used to compare two groups.

## Data Availability

All data are available in the manuscript or through linked online databases with analyzed and raw data sets. See methods for links to raw sequencing data deposited into GEO.

## SUPPLEMENTAL MATERIAL

Table S1 iai.00136-26-s0001.xlsx Normalized counts for RNAseq analysis in Figures 3 and 7.

Table S2 iai.00136-26-s0002.xlsx Bioinformatic analysis underlying Figures 3 and 7 clustering.

ASM does not own the copyrights to Supplemental Material that may be linked to, or accessed through, an article. The authors have granted ASM a non-exclusive, world-wide license to publish the Supplemental Material files. Please contact the corresponding author directly for reuse.

## References

[1] Johansen MD, Herrmann JL, Kremer L. 2020. Non-tuberculous mycobacteria and the rise of Mycobacterium abscessus. Nat Rev Microbiol 18:392–407. doi:10.1038/s41579-020-0331-1 32086501

[2] Sharma SK, Upadhyay V. 2020. Epidemiology, diagnosis & treatment of non-tuberculous mycobacterial diseases. Indian J Med Res 152:185–226. doi:10.4103/ijmr.IJMR_902_20 33107481 PMC7881820

[3] Prevots DR, Shaw PA, Strickland D, Jackson LA, Raebel MA, Blosky MA, Montes de Oca R, Shea YR, Seitz AE, Holland SM, et al.. 2010. Nontuberculous mycobacterial lung disease prevalence at four integrated health care delivery systems. Am J Respir Crit Care Med 182:970–976. doi:10.1164/rccm.201002-0310OC 20538958 PMC2970866

[4] Victoria L, Gupta A, Gómez JL, Robledo J. 2021. Mycobacterium abscessus complex: a review of recent developments in an emerging pathogen. Front Cell Infect Microbiol 11:659997. doi:10.3389/fcimb.2021.659997 33981630 PMC8108695

[5] Roux AL, Catherinot E, Ripoll F, Soismier N, Macheras E, Ravilly S, Bellis G, Vibet M-A, Le Roux E, Lemonnier L, et al.. 2009. Multicenter study of prevalence of nontuberculous mycobacteria in patients with cystic fibrosis in France. J Clin Microbiol 47:4124–4128. doi:10.1128/JCM.01257-09 19846643 PMC2786646

[6] Olivier KN, Weber DJ, Wallace RJ, Faiz AR, Lee J-H, Zhang Y, Brown-Elliot BA, Handler A, Wilson RW, Schechter MS, et al.. 2003. Nontuberculous mycobacteria. i: multicenter prevalence study in cystic fibrosis. Am J Respir Crit Care Med 167:828–834. doi:10.1164/rccm.200207-678OC 12433668

[7] De Rose V, Molloy K, Gohy S, Pilette C, Greene CM. 2018. Airway epithelium dysfunction in cystic fibrosis and COPD. Mediators Inflamm 2018:1309746. doi:10.1155/2018/1309746 29849481 PMC5911336

[8] Fahy JV, Dickey BF. 2010. Airway mucus function and dysfunction. N Engl J Med 363:2233–2247. doi:10.1056/NEJMra0910061 21121836 PMC4048736

[9] Daley CL, Iaccarino JM, Lange C, Cambau E, Wallace RJ, Andrejak C, Böttger EC, Brozek J, Griffith DE, Guglielmetti L, et al.. 2020. Treatment of nontuberculous mycobacterial pulmonary disease: an official ATS/ERS/ESCMID/IDSA clinical practice guideline. Clin Infect Dis 71:905–913. doi:10.1093/cid/ciaa1125 32797222 PMC7768745

[10] Nessar R, Cambau E, Reyrat JM, Murray A, Gicquel B. 2012. Mycobacterium abscessus: a new antibiotic nightmare. J Antimicrob Chemother 67:810–818. doi:10.1093/jac/dkr578 22290346

[11] Jarand J, Levin A, Zhang L, Huitt G, Mitchell JD, Daley CL. 2011. Clinical and microbiologic outcomes in patients receiving treatment for Mycobacterium abscessus pulmonary disease. Clin Infect Dis 52:565–571. doi:10.1093/cid/ciq237 21292659

[12] Kreda SM, Davis CW, Rose MC. 2012. CFTR, mucins, and mucus obstruction in cystic fibrosis. Cold Spring Harb Perspect Med 2:a009589. doi:10.1101/cshperspect.a009589 22951447 PMC3426818

[13] Moni SS, Al Basheer A. 2022. Molecular targets for cystic fibrosis and therapeutic potential of monoclonal antibodies. Saudi Pharm J 30:1736–1747. doi:10.1016/j.jsps.2022.10.002 36601503 PMC9805982

[14] Murray PJ, Wynn TA. 2011. Protective and pathogenic functions of macrophage subsets. Nat Rev Immunol 11:723–737. doi:10.1038/nri3073 21997792 PMC3422549

[15] Byrne AJ, Mathie SA, Gregory LG, Lloyd CM. 2015. Pulmonary macrophages: key players in the innate defence of the airways. Thorax 70:1189–1196. doi:10.1136/thoraxjnl-2015-207020 26286722

[16] Croasdell A, Duffney PF, Kim N, Lacy SH, Sime PJ, Phipps RP. 2015. PPARγ and the innate immune system mediate the resolution of inflammation. PPAR Res 2015:549691. doi:10.1155/2015/549691 26713087 PMC4680113

[17] McQuattie-Pimentel AC, Ren Z, Joshi N, Watanabe S, Stoeger T, Chi M, Lu Z, Sichizya L, Aillon RP, Chen C-I, et al.. 2021. The lung microenvironment shapes a dysfunctional response of alveolar macrophages in aging. J Clin Invest 131:e140299. doi:10.1172/JCI140299 33586677 PMC7919859

[18] Nakayama Y, Sasai M, Kuratani A, Okamoto M, Okuzaki D, Yamamoto K, Ono C, Yamaguchi M, Kawabata S, Shinjyo N, et al.. 2025. Targeted labeling and depletion of alveolar macrophages using VeDTR mouse technology. iScience 28:111975. doi:10.1016/j.isci.2025.111975 40060898 PMC11889737

[19] Ankley LM, Conner KN, Vielma TE, Godfrey JJ, Thapa M, Olive AJ. 2024. GSK3α/β restrain IFN-γ-inducible costimulatory molecule expression in alveolar macrophages, limiting CD4+ t cell activation. Immunohorizons 8:147–162. doi:10.4049/immunohorizons.2300107 38345473 PMC10916365

[20] Srivastava S, Ernst JD. 2013. Cutting edge: direct recognition of infected cells by CD4 t cells is required for control of intracellular Mycobacterium tuberculosis in vivo. J Immunol 191:1016–1020. doi:10.4049/jimmunol.1301236 23817429 PMC3725655

[21] Huang L, Nazarova EV, Tan S, Liu Y, Russell DG. 2018. Growth of Mycobacterium tuberculosis in vivo segregates with host macrophage metabolism and ontogeny. J Exp Med 215:1135–1152. doi:10.1084/jem.20172020 29500179 PMC5881470

[22] Pisu D, Huang L, Grenier JK, Russell DG. 2020. Dual RNA-seq of mtb-infected macrophages in vivo reveals ontologically distinct host-pathogen interactions. Cell Rep 30:335–350. doi:10.1016/j.celrep.2019.12.033 31940480 PMC7032562

[23] Goulding J, Snelgrove R, Saldana J, Didierlaurent A, Cavanagh M, Gwyer E, Wales J, Wissinger EL, Hussell T. 2007. Respiratory infections: do we ever recover? Proc Am Thorac Soc 4:618–625. doi:10.1513/pats.200706-066TH 18073393 PMC2647650

[24] Schneider WM, Chevillotte MD, Rice CM. 2014. Interferon-stimulated genes: a complex web of host defenses. Annu Rev Immunol 32:513–545. doi:10.1146/annurev-immunol-032713-120231 24555472 PMC4313732

[25] Rosain J, Kong X-F, Martinez-Barricarte R, Oleaga-Quintas C, Ramirez-Alejo N, Markle J, Okada S, Boisson-Dupuis S, Casanova J-L, Bustamante J. 2019. Mendelian susceptibility to mycobacterial disease: 2014-2018 update. Immunol Cell Biol 97:360–367. doi:10.1111/imcb.12210 30264912 PMC6438774

[26] Tau G, Rothman P. 1999. Biologic functions of the IFN-gamma receptors. Allergy 54:1233–1251. doi:10.1034/j.1398-9995.1999.00099.x 10688427 PMC4154595

[27] Kak G, Raza M, Tiwari BK. 2018. Interferon-gamma (IFN-γ): exploring its implications in infectious diseases. Biomol Concepts 9:64–79. doi:10.1515/bmc-2018-0007 29856726

[28] Thiel BA, Lundberg KC, Schlatzer D, Jarvela J, Li Q, Shaw R, Reba SM, Fletcher S, Beckloff SE, Chance MR, et al.. 2024. Human alveolar macrophages display marked hypo-responsiveness to IFN-γ in both proteomic and gene expression analysis. PLoS One 19:e0295312. doi:10.1371/journal.pone.0295312 38300916 PMC10833554

[29] Ahn JH, Park JY, Kim DY, Lee TS, Jung DH, Kim YJ, Lee YJ, Lee YJ, Seo IS, Song EJ, et al.. 2021. Type I interferons are involved in the intracellular growth control of Mycobacterium abscessus by mediating NOD2-induced production of nitric oxide in macrophages. Front Immunol 12:738070. doi:10.3389/fimmu.2021.738070 34777348 PMC8581665

[30] Gilliland HN, Beckman OK, Olive AJ. 2023. A genome-wide screen in macrophages defines host genes regulating the uptake of Mycobacterium abscessus. mSphere 8:e0066322. doi:10.1128/msphere.00663-22 36794958 PMC10117111

[31] Laencina L, Dubois V, Le Moigne V, Viljoen A, Majlessi L, Pritchard J, Bernut A, Piel L, Roux A-L, Gaillard J-L, et al.. 2018. Identification of genes required for Mycobacterium abscessus growth in vivo with a prominent role of the ESX-4 locus. Proc Natl Acad Sci USA 115:E1002–E1011. doi:10.1073/pnas.1713195115 29343644 PMC5798338

[32] Le Moigne V, Belon C, Goulard C, Accard G, Bernut A, Pitard B, Gaillard J-L, Kremer L, Herrmann J-L, Blanc-Potard A-B. 2016. MgtC as a host-induced factor and vaccine candidate against Mycobacterium abscessus infection. Infect Immun 84:2895–2903. doi:10.1128/IAI.00359-16 27481243 PMC5038086

[33] Means TK, Jones BW, Schromm AB, Shurtleff BA, Smith JA, Keane J, Golenbock DT, Vogel SN, Fenton MJ. 2001. Differential effects of a toll-like receptor antagonist on Mycobacterium tuberculosis-induced macrophage responses. J Immunol 166:4074–4082. doi:10.4049/jimmunol.166.6.4074 11238656

[34] Bernut A, Nguyen-Chi M, Halloum I, Herrmann JL, Lutfalla G, Kremer L. 2016. Mycobacterium abscessus-Induced granuloma formation is strictly dependent on tnf signaling and neutrophil trafficking. PLoS Pathog 12:e1005986. doi:10.1371/journal.ppat.1005986 27806130 PMC5091842

[35] Bernut Audrey, Herrmann J-L, Kissa K, Dubremetz J-F, Gaillard J-L, Lutfalla G, Kremer L. 2014. Mycobacterium abscessus cording prevents phagocytosis and promotes abscess formation. Proc Natl Acad Sci USA 111:E943–52. doi:10.1073/pnas.1321390111 24567393 PMC3956181

[36] Thomas ST, Wierenga KA, Pestka JJ, Olive AJ. 2022. Fetal liver-derived alveolar-like macrophages: a self-replicating ex vivo model of alveolar macrophages for functional genetic studies. Immunohorizons 6:156–169. doi:10.4049/immunohorizons.2200011 35193942 PMC10217771

[37] Fejer G, Wegner MD, Györy I, Cohen I, Engelhard P, Voronov E, Manke T, Ruzsics Z, Dölken L, Prazeres da Costa O, et al.. 2013. Nontransformed, GM-CSF-dependent macrophage lines are a unique model to study tissue macrophage functions. Proc Natl Acad Sci USA 110:E2191–8. doi:10.1073/pnas.1302877110 23708119 PMC3683787

[38] Luo M, Lai W, He Z, Wu L. 2021. Development of an optimized culture system for generating mouse alveolar macrophage-like cells. J Immunol 207:1683–1693. doi:10.4049/jimmunol.2100185 34400525

[39] Guilliams M, De Kleer I, Henri S, Post S, Vanhoutte L, De Prijck S, Deswarte K, Malissen B, Hammad H, Lambrecht BN. 2013. Alveolar macrophages develop from fetal monocytes that differentiate into long-lived cells in the first week of life via GM-CSF. J Exp Med 210:1977–1992. doi:10.1084/jem.20131199 24043763 PMC3782041

[40] Yu X, Buttgereit A, Lelios I, Utz SG, Cansever D, Becher B, Greter M. 2017. The cytokine TGF-β promotes the development and homeostasis of alveolar macrophages. Immunity 47:903–912. doi:10.1016/j.immuni.2017.10.007 29126797

[41] Thomas SM, McGee AP, Vielma TE, Ankley LM, Rapp AW, Conner KN, LeSage F, Tanner CD, Scheeres EC, Obar JJ, Olive AJ. 2026. TGFβ primes alveolar-like macrophages to induce type I IFN following TLR2 activation. J Immunol 215:vkag090. doi:10.1093/jimmun/vkag090 42152611 PMC13183718

[42] Kiritsy MC, McCann K, Mott D, Holland SM, Behar SM, Sassetti CM, Olive AJ. 2021. Mitochondrial respiration contributes to the interferon gamma response in antigen-presenting cells. eLife 10. doi:10.7554/eLife.65109 PMC8598164 34726598

[43] Rothchild AC, Olson GS, Nemeth J, Amon LM, Mai D, Gold ES, Diercks AH, Aderem A. 2019. Alveolar macrophages generate a noncanonical NRF2-driven transcriptional response to Mycobacterium tuberculosis in vivo. Sci Immunol 4:eaaw6693. doi:10.1126/sciimmunol.aaw6693 31350281 PMC6910245

[44] Bai X, Feldman NE, Chmura K, Ovrutsky AR, Su W-L, Griffin L, Pyeon D, McGibney MT, Strand MJ, Numata M, et al.. 2013. Inhibition of nuclear factor-kappa b activation decreases survival of Mycobacterium tuberculosis in human macrophages. PLoS One 8:e61925. doi:10.1371/journal.pone.0061925 23634218 PMC3636238

[45] Abdelaal HFM, Chan ED, Young L, Baldwin SL, Coler RN. 2022. Mycobacterium abscessus: it’s complex. Microorganisms 10:1454. doi:10.3390/microorganisms10071454 35889173 PMC9316637

[46] Braverman J, Stanley SA. 2017. Nitric oxide modulates macrophage responses to Mycobacterium tuberculosis infection through activation of HIF-1α and repression of NF-κB. J Immunol 199:1805–1816. doi:10.4049/jimmunol.1700515 28754681 PMC5568107

[47] Braverman J, Sogi KM, Benjamin D, Nomura DK, Stanley SA. 2016. HIF-1α Is an essential mediator of IFN-γ-dependent immunity to Mycobacterium tuberculosis. J Immunol 197:1287–1297. doi:10.4049/jimmunol.1600266 27430718 PMC4976004

[48] Mishra BB, Lovewell RR, Olive AJ, Zhang G, Wang W, Eugenin E, Smith CM, Phuah JY, Long JE, Dubuke ML, et al.. 2017. Nitric oxide prevents a pathogen-permissive granulocytic inflammation during tuberculosis. Nat Microbiol 2:17072. doi:10.1038/nmicrobiol.2017.72 28504669 PMC5461879

[49] Jantsch J, Wiese M, Schödel J, Castiglione K, Gläsner J, Kolbe S, Mole D, Schleicher U, Eckardt K-U, Hensel M, et al.. 2011. Toll-like receptor activation and hypoxia use distinct signaling pathways to stabilize hypoxia-inducible factor 1α (HIF1A) and result in differential HIF1A-dependent gene expression. J Leukoc Biol 90:551–562. doi:10.1189/jlb.1210683 21685248

[50] Li C, Wang Y, Li Y, Yu Q, Jin X, Wang X, Jia A, Hu Y, Han L, Wang J, et al.. 2018. HIF1α-dependent glycolysis promotes macrophage functional activities in protecting against bacterial and fungal infection. Sci Rep 8. doi:10.1038/s41598-018-22039-9 PMC5827022 29483608

[51] Wang T, Liu H, Lian G, Zhang S-Y, Wang X, Jiang C. 2017. HIF1α-induced glycolysis metabolism is essential to the activation of inflammatory macrophages. Mediators Inflamm 2017:9029327. doi:10.1155/2017/9029327 29386753 PMC5745720

[52] Roux AL, Viljoen A, Bah A, Simeone R, Bernut A, Laencina L, Deramaudt T, Rottman M, Gaillard JL, Majlessi L, et al.. 2016. The distinct fate of smooth and rough Mycobacterium abscessus variants inside macrophages. Open Biol 6:160185. doi:10.1098/rsob.160185 27906132 PMC5133439

[53] Kim BR, Kim BJ, Kook YH, Kim BJ. 2019. Phagosome escape of rough Mycobacterium abscessus strains in murine macrophage via phagosomal rupture can lead to type i interferon production and their cell-to-cell spread. Front Immunol 10:125. doi:10.3389/fimmu.2019.00125 30766538 PMC6365470

[54] Gutiérrez AV, Viljoen A, Ghigo E, Herrmann J-L, Kremer L. 2018. Glycopeptidolipids, a double-edged sword of the Mycobacterium abscessus complex. Front Microbiol 9:1145. doi:10.3389/fmicb.2018.01145 29922253 PMC5996870

[55] Howard ST, Rhoades E, Recht J, Pang X, Alsup A, Kolter R, Lyons CR, Byrd TF. 2006. Spontaneous reversion of Mycobacterium abscessus from a smooth to a rough morphotype is associated with reduced expression of glycopeptidolipid and reacquisition of an invasive phenotype. Microbiology (Reading, Engl) 152:1581–1590. doi:10.1099/mic.0.28625-0 16735722

[56] Bernut A, Dupont C, Ogryzko NV, Neyret A, Herrmann J-L, Floto RA, Renshaw SA, Kremer L. 2019. CFTR protects against Mycobacterium abscessus Infection by fine-tuning host oxidative defenses. Cell Rep 26:1828–1840. doi:10.1016/j.celrep.2019.01.071 30759393 PMC7618368

[57] Rhoades ER, Archambault AS, Greendyke R, Hsu FF, Streeter C, Byrd TF. 2009. Mycobacterium abscessus glycopeptidolipids mask underlying cell wall phosphatidyl-myo-inositol mannosides blocking induction of human macrophage TNF-alpha by preventing interaction with TLR2. J Immunol 183:1997–2007. doi:10.4049/jimmunol.0802181 19596998

[58] Kiritsy MC, Ankley LM, Trombley J, Huizinga GP, Lord AE, Orning P, Elling R, Fitzgerald KA, Olive AJ. 2021. A genetic screen in macrophages identifies new regulators of IFNγ-inducible MHCII that contribute to T cell activation. eLife 10:e65110. doi:10.7554/eLife.65110 34747695 PMC8598162

[59] Roberts AW, Popov LM, Mitchell G, Ching KL, Licht DJ, Golovkine G, Barton GM, Cox JS. 2019. Cas9^+^ conditionally-immortalized macrophages as a tool for bacterial pathogenesis and beyond. eLife 8:e45957. doi:10.7554/eLife.45957 31204998 PMC6579556

[60] Johnson BK, Thomas SM, Olive AJ, Abramovitch RB. 2021. Macrophage infection models for Mycobacterium tuberculosis. Methods Mol Biol 2314:167–182. doi:10.1007/978-1-0716-1460-0_6 34235652

[61] Köster J, Rahmann S. 2012. Snakemake--a scalable bioinformatics workflow engine. Bioinformatics 28:2520–2522. doi:10.1093/bioinformatics/bts480 22908215

[62] Andrews S. 2010. FastQC: a quality control tool for high throughput sequence data: babraham bioinformatics. Available from: http://www.bioinformatics.babraham.ac.uk/projects/fastqc

[63] Langmead B, Salzberg SL. 2012. Fast gapped-read alignment with bowtie 2. Nat Methods 9:357–359. doi:10.1038/nmeth.1923 22388286 PMC3322381

[64] Liao Y, Smyth GK, Shi W. 2014. featureCounts: an efficient general purpose program for assigning sequence reads to genomic features. Bioinformatics 30:923–930. doi:10.1093/bioinformatics/btt656 24227677

[65] Love MI, Huber W, Anders S. 2014. Moderated estimation of fold change and dispersion for RNA-seq data with DESeq2. Genome Biol 15:550. doi:10.1186/s13059-014-0550-8 25516281 PMC4302049

[66] H W. 2016. Ggplot2: elegant graphics for data analysis. Springer-Verlag, New York.

[67] Chen H, Boutros PC. 2011. Venndiagram: a package for the generation of highly-customizable venn and euler diagrams in r. BMC Bioinformatics 12:35. doi:10.1186/1471-2105-12-35 21269502 PMC3041657

[68] Abu-Jamous B, Kelly S. 2018. Clust: automatic extraction of optimal co-expressed gene clusters from gene expression data. Genome Biol 19:172. doi:10.1186/s13059-018-1536-8 30359297 PMC6203272

[69] Reimand J, Kull M, Peterson H, Hansen J, Vilo J. 2007. g:Profiler--a web-based toolset for functional profiling of gene lists from large-scale experiments. Nucleic Acids Res 35:W193–200. doi:10.1093/nar/gkm226 17478515 PMC1933153

